# Effects of Microbiota Imbalance in Anxiety and Eating Disorders: Probiotics as Novel Therapeutic Approaches

**DOI:** 10.3390/ijms22052351

**Published:** 2021-02-26

**Authors:** Elisabet Navarro-Tapia, Laura Almeida-Toledano, Giorgia Sebastiani, Mariona Serra-Delgado, Óscar García-Algar, Vicente Andreu-Fernández

**Affiliations:** 1 Grup de Recerca Infancia i Entorn (GRIE), Institut d′investigacions Biomèdiques August Pi i Sunyer (IDIBAPS), 08036 Barcelona, Spain; elisabetnavarrotapia@gmail.com (E.N.-T.); ogarciaa@clinic.cat (O.G.-A); 2 Institut de Recerca Sant Joan de Déu, 08950 Esplugues de Llobregat, Spain; lalmeida@sjdhospitalbarcelona.org (L.A.-T.); mserrad@sjdhospitalbarcelona.org (M.S.-D.); 3 BCNatal, Fetal Medicine Research Center (Hospital Sant Joan de Déu and Hospital Clínic), University of Barcelona, 08950 Barcelona, Spain; 4 Department of Neonatology, Hospital Clínic-Maternitat, ICGON, BCNatal, 08028 Barcelona, Spain; gsebasti@clinic.cat; 5 Department of Health, Valencian International University (VIU), 46002 Valencia, Spain

**Keywords:** probiotics, gut microbiota, dysbiosis, gut–brain axis, anorexia nervosa, bulimia nervosa, binge-eating disorder, anxiety

## Abstract

Anxiety and eating disorders produce a physiological imbalance that triggers alterations in the abundance and composition of gut microbiota. Moreover, the gut–brain axis can be altered by several factors such as diet, lifestyle, infections, and antibiotic treatment. Diet alterations generate gut dysbiosis, which affects immune system responses, inflammation mechanisms, the intestinal permeability, as well as the production of short chain fatty acids and neurotransmitters by gut microbiota, which are essential to the correct function of neurological processes. Recent studies indicated that patients with generalized anxiety or eating disorders (anorexia nervosa, bulimia nervosa, and binge-eating disorders) show a specific profile of gut microbiota, and this imbalance can be partially restored after a single or multi-strain probiotic supplementation. Following the PRISMA methodology, the current review addresses the main microbial signatures observed in patients with generalized anxiety and/or eating disorders as well as the importance of probiotics as a preventive or a therapeutic tool in these pathologies.

## 1. Introduction

The interest in mental health has increased in recent years. Anxiety and mood disorders are associated with many disabilities and individual suffering. The overall prevalence of anxiety ranges from 5% to 30%, and of mood disorders from 5% to 15% [1,2]. Moreover, the COVID-19 pandemic has increased these percentages, as demonstrated by a recent meta-analysis conducted in the general population and in healthcare workers, showing a prevalence for anxiety of 30% and 23.2%, respectively [3,4]. Furthermore, there is a close relationship between eating disorders (EDs) and anxiety. The most prevalent EDs are anorexia nervosa (AN), bulimia nervosa (BN), and binge eating disorder (BED), with a lifetime prevalence of 0.48%, 0.51%, and 1.12%, respectively. All of them usually start between 10 and 20 years old, predominantly in females [5]. The lifetime prevalence of any ED is 2.5% in the European population, these patients have a prevalence of 33–40% of any anxiety disorder and 19–50% of any mood disorder [5]. However, the pathophysiology of anxiety and EDs is poorly understood due to the complexity of analyzing the genetic, metabolic, and environmental factors that are involved in the appearance of these disorders.

The altered neural mechanisms in EDs are mainly related to the reward, behavioral control, and decision-making paths. The current bibliography suggests that amygdala, hippocampus, and medial prefrontal cortex are functionally compromised in anxiety disorder [6]. These brain regions are involved in the generation and regulation of emotions and fear. In anorexia and BN, there is a greater connectivity between insula, orbitofrontal cortex, and ventral striatum, but lower connectivity from orbitofrontal cortex and amygdala to the hypothalamus [7]. In BED, there is a diminished activity in the ventromedial prefrontal cortex, inferior frontal gyrus, and insula [8], areas that are involved in self-regulation and impulse control. Moreover, the alteration of the dopamine pathway is an important contributor to the developing of any ED [8,9,10]. In anorexia and BN, harm avoidance mechanisms related to serotonin receptor availability and to dopamine receptor binding are also altered [11]. Otherwise, the neuropeptides that manage the signal of hunger (ghrelin) and satiety (leptin) interact with the mesolimbic dopamine system, being altered in EDs. Leptin is an anorexigenic peptide released from adipose tissue that is diminished in AN. Ghrelin peptide with orexigenic functions is elevated in AN and does not respond correctly after food intake [12]. Sensitivity to insulin (anorexigenic pancreatic hormone) is also increased in AN [13]. Stress, hyperactivity, and appetite are also modulated by the cortisol awakening response of hypothalamic–pituitary–adrenal (HPA) axis, being imbalanced in anxiety and AN [14].

One of the factors that influence the pathophysiology of anxiety and EDs is the composition of gut microbiota due to the strong association between the microbial signature and the brain function. The gut microbiota includes the phyla *Firmicutes* (including *Lactobacillus, Enterococcus*, and *Clostridium* genera) and *Bacteroidetes* (including *Bacteroides* genus), which represent more than 90% of the intestinal community in healthy adults as well as *Actinobacteria* and *Proteobacteria* [15,16]. The gut–brain axis refers to the bidirectional interaction between the gut microbiota and the central nervous system (CNS). This interaction has been shown an increasing interest in recent years due to the harmful effects of dysbiosis on brain function. Gut bacteria interact with the CNS by synthetizing neurotransmitters such as serotonin, dopamine, gamma aminobutyric acid (GABA), acetylcholine, and glutamate and respond to hormones. Moreover, gut microbial diversity is also associated with dysregulation of appetite due to ability to influence the intestinal satiety pathways [17].

Diet influences the microbial composition and richness. The fermentation of indigestible carbohydrates by the colon microbiota produce short-chain fatty acids (SCFAs), such as acetate, propionate, and butyrate, implicated on the maintenance of homeostasis, the regulation of appetite, and anti-inflammatory processes [18]. Diet alterations can generate an imbalance of microbial diversity and richness (alpha-diversity), which reduces gut *Firmicutes* and increases *Bacteroides* phyla [19]. Moreover, alterations of gut microbiota decrease the intake of calories from the diet [20], altering the immunological response. The innate immune system is activated under dysbiosis by the increase of bacterial lipopolysaccharides (LPS). These endotoxins trigger the release of pro-inflammatory cytokines, such as interleukin-6 (IL-6) and tumor necrosis factor alpha (TNF-α) in plasma, and downregulate synaptic proteins through the subdiaphragmatic vague nerve [21]. Moreover, recent studies show that the gut microbiota modulates the reactivity of the HPA axis, which influences the endocrine pathway. Therefore, its imbalance can produce abnormal glucocorticoid levels and promote behavioral changes [22].

Due to the role of gut microbiota on modulation of neuronal circuits through the gut–brain axis, the analysis of microbial profiles in patients with anxiety and/or EDs is of vital importance. The knowledge of the changes in the microbial communities of these patients could help in the development of novel therapeutic tools based on the modulation of the microbiome. 

## 2. Results

### 2.1. Role of Microbiota in Anxiety

Anxiety is considered one of the most frequent mental disorders and is highly comorbid with other mental disorders and EDs [23,24]. A 2004 study found that about two-thirds of 672 individuals with AN, BN, or both had one or more lifetime anxiety disorders [25]. Moreover, anxiety disorders, especially obsessive–compulsive disorder (OCD) in childhood, could be a risk factor for EDs later in life [26]. Although the etiology of anxiety has traditionally been focused on genetic factors, evidences indicate that the gut microbiota and its metabolites are closely linked to the host’s central nervous system though a bidirectional communication. The microbiome plays an important role in the programming early in life of the HPA axis, a primary component of the stress response, and its reactivity in adult life [27]. Recent studies showed that prenatal stress in mice is related to alterations in the microbiome, cytokines levels, and reduction of the brain derived neurotrophic factor (BDNF) in the offspring, suggesting a link between the microbiome at early stages and adult behavioral changes [28]. Moreover, a transfer of vaginal microbiome from stressed mothers to offspring that were not stressed resulted in higher corticosterone levels and an altered gene expression in the para-ventricular nucleus (PVN) of the hypothalamus after stress exposure in adulthood [29].

Stress decreases specific intestinal epithelial tight junction proteins such as claudin 1 (CLDN1) and damages the integrity of the gut epithelium [30], altering gut motility, secretions, and mucin production [31,32]. These changes in the habitat of resident bacteria will promote changes in microbial composition and intestinal permeability. One of the most relevant is the translocation of certain gram-negative bacteria and allergens to the bloodstream that will trigger the inflammasome signaling and neuroinflammatory processes [33,34,35]. Inflammatory and neuromodulatory cytokines in the systemic circulation will reach the brain due to an altered permeability of the blood–brain barrier (BBB), enhancing mood disorders such as anxiety [36,37]. Therefore, an altered intestinal microbiota can alter behavior, immunity, and endocrinology, and has been associated with central nervous disorders, such as autism, and depression or anxiety behaviors. 

Preclinical models highlight how gut microbiota disturbances due to antibiotics, delivery mode, or fecal microbiota transplantation (FMT) from AN patients correlated with anxiety-like behaviors [38,39,40,41]. Lach et al., assessed the long-term effects of transient gut microbiota depletion using a cocktail of five antibiotics in mice. Results showed a decrease of members of the family *Lachnospiraceae* and *Bacteroides* genus, which are also associated with anxiety in humans [42]. Furthermore, expression of genes involved in neurotransmission (such as *Gabra1*, *Gabbr1*, *Npy,* or *Nr3c1*), SCFA, and tight-junction proteins were more drastically affected during adolescence than adulthood [43]. These results highlight the consequences of gut microbiota manipulation during adolescence in mood disorders and the need for this kind of study in humans. 

Given the role of gut microbiota in the gut–brain axis due its capacity to produce neurotransmitters (e.g., GABA by *Bifidobacterium* [44]) and its precursors as well as cytokines, BDNF, and SCFA [45], several efforts have been carried out to establish a microbiological signature in individuals with anxiety disorders. Unfortunately, only a few studies in humans achieved the establishment of taxonomic differences between anxiety disorders relative to controls [42,46,47]. In all three studies analyzed, participants with generalized anxiety disorders (GAD) showed lower microbial richness, measured by the number of observed operational taxonomic units (OTUs), compared to controls. In a cross-sectional study of 36 heathy controls and 40 patients with GAD, Jiang et al., observed a significant decrease of *Firmicutes* spp., specially *Butyricicoccus, Lachnospira,* and the anti-inflammatory commensal bacterium *Faecalibacterium* in GAD patients. Conversely, Fusobacteria, a pathobiont with invasive and proinflammatory properties, and *Bacteroidetes* spp. increased significantly. Interestingly, the abundance of *Ruminococcus gnavus,* related to Crohn’s disease and the producer of an inflammatory polysaccharide [48], was significantly higher in GAD patients. This study showed that gender or remission had no effect on the relative abundance of the different taxa; however, the medication did. An enrichment of *Escherichia–Shigella* in treatment-naïve GAD was observed after a subgroup analysis [42].

Another study in a Chinese population with GAD published a year later also showed a greater enrichment in *Escherichia*–*Shigella* and *Bacteroides* [47]. Other groups such as *Enterobacteriaceae*, *Enterobacteriales*, and *Bacteroidaceae* were also increased in these patients, highlighting again the relationship between the presence of pathogens in the gut and anxiety. On the contrary, *Firmicutes*, *Dialister*, *Prevotellaceae*, *Coprococcus*, *Clostridium innocuum* group, *Buchnera*, *Tenericutes*, *Mollicutes,* and *Acinetobacter* were more abundant in healthy controls. Interestingly, abundances of *Bacteroides* and *Escherichia*–*Shigella* were positively associated with anxiety severity, whereas *Eubacterium*_*coprostanoligenes*_group, *Ruminococcaceae*_UCG-014, and *Prevotella*_9 correlated positively with anxiety reduction. These authors observed association between gender and the gut-microbial community composition in patients with GAD (contrary to Jiang et al.). Unfortunately, they did not examine the effects of drugs on gut microbiota profiles. Only two case-control studies have been carried out for the study of a microbiological signature related exclusively to GAD, both being carried out in the Asian population. Only one study was performed in the Caucasian population, although the subjects exclusively diagnosed with anxiety was very low (*n* = 8) and all females were under medication [46]. In this study, Mason et al., associated negatively the abundance of *Eubacterium rectale*/*Closteridium* group (*Clostridium* cluster XIVa) and *Clostridium leptum* group (*Clostridium* cluster IV) with the severity of anxiety (measured as GAD-7 score). Neither α-diversity nor β-diversity were significantly associated with this score, contrary to the other two studies where GAD was associated with a lower fecal bacterial α-diversity. Likewise, the abundance of *Bacteroides* was also inconsistent with previous studies, since, in this case, reductions in *Bacteroides* were associated with the presence of anxiety. However, due to the bias in sex and the limited sample size, these conclusions should be analyzed with caution [46]. 

Overall these studies identified an imbalance of microbial communities in GAD patients, suggesting that microbiome modulation may be a preventive and therapeutic tool for anxiety.

### 2.2. Microbiota and Anorexia Nervosa

The intestinal microbiota plays a crucial role in metabolic function, immunomodulation, and weight regulation. AN represents a severe mental illness characterized by severe weight loss associated with biochemical, metabolic, and immunologic disabilities, as well as high mortality rates [49].

The gut microbiota exerts a critical role in weight gain and energy intake from the diet. Dietary changes may also provoke an intense microbiota shift [50]. Furthermore, the microbiota impacts satiety pathways through interaction with peptide signaling and may be included in the etiology of AN [51]. The interactions between microbiota and the CNS are regulated by neuroendocrine and metabolic pathways, adjusting the balance between anorexigenic hormones like alpha melanocyte stimulating hormone (αMSH) and orexigenic peptides (ghrelins, leptine, and orexin) originated from the gastrointestinal tract [52]. *Bifidobacterium* spp. and *Lactobacillus* spp. produce GABA, related to anxiety control; *Enterococcus* spp., *Escherichia* spp., *Streptococcus* spp., and *Candida* spp. produce serotonin, a neurotransmitter involved in mood regulation, as well as dopamine produced by *Bacillus* spp. [53,54]. In general, gram-negative bacteria produces bacterial lipopolysaccharides, involved in the regulation of food intake through the activation of the enteroendocrine cells expressing toll-like receptors (TLRs) [55]. An imbalance of such molecules may impair feeding behaviors and weight loss [56]. Moreover, disorders in microbiota composition have been linked to anxiety and depression typical of AN patients, and psychological stress may lead to microbial translocation that enhance gut inflammation [57].

The first case studies in people with AN showed 11 new bacterial species classified in Firmicutes (Soleaferrea massiliensis, Stoquefichus massiliensis, Dorea massiliensis, Holdemania massiliensis, Clostridium anorexicus, Clostridium anorexicamassiliense, Bacillus marseilloanorexicus), Bacteroidetes (Bacteroides timonensis, Alistipes marseilloanorexicus), and Actinobacteria (Streptomyces massiliensis, Blastococcus massiliensis) phyla and four new micro-eukaryote species (Tetratrichomonas sp., Aspergillus ruber, Penicillium solitum, and Cladosporium bruhnei), respectively, in a single fecal sample, demonstrating an abnormal state in the intestinal tract of people with AN [58,59] (Table 1).

Complex carbohydrates are metabolized by intestinal microorganisms into SCFA with neuroactive involvement (n-butyrate, acetate, and propionate) [60,61]. Recent clinical studies (Table 1) conducted in the AN population showed that the total amount of bacteria including *Clostridium coccoides*, *Clostridium leptum*, and *Bacteroides fragilis* was significantly decreased in AN patients [62]. In addition, *Streptococcus*, *Lactobacillus plantarum*, and the genera butyrate-producing *Roseburia*, carbohidrate-fermenter *Ruminococcus*, and *Clostridium*, belonging to *Firmicutes*, were reduced with the subsequent reduced acetic, propionic, and butyrate acid concentration in the feces of patients with AN [62,63,64,65]. Propionate exhibited a positive correlation with insulin concentrations and with the relative depletion of the propionate producer *Roseburia inulinivorans*, whereas butyrate levels were negatively correlated with anxiety and depression [63]. This may explain the reduced insulin levels and the increased anxiety in AN individuals. Additionally, a reduction of the butyrate-producing *Roseburia* spp. was identified in AN compared with healthy controls leading to elevated branched-chain fatty acid concentrations and products of protein fermentation, which may impair gut physiology and motility [66,67]. In contrast, mucin-degraders and members of *Clostridium* clusters I, XI, and XVIII; *Actinobacteria* (mainly *Bifidobacteria*) [66], *Enterobacteriaceae*, and the methan-producing archeon *Methanobrevibacter smithii* [63], as well as *Coriobacteriaceae*, were increased in AN compared with healthy controls. *Methanobrevibacter smithii* improves the efficiency of microbial fermentation, and its richness optimizes calorie extraction from a diet with very low calorie content. The development of *Methanobrevibacter* in AN patients might be associated with an adaptive mechanism to optimize the absorption of a hypocaloric diet [64]. The nutrient-deficient environment, together with a delayed colonic transit in AN patients, favors the increase of mucin-degrading microorganisms. This will contribute to a disrupted gut barrier and a chronic state of low-grade inflammation, exacerbating the disease [68]. Therefore, the microbiota profile has been linked to gut inflammation and impaired structure of epithelial layer [69], as demonstrated by the increased levels of IL-6 found in AN patients and IL-6 and IL-1 α in obese individuals [70]. LPS also produces an increase in blood–brain barrier permeability with the elevation of plasma circulating cytokines responsible for the anorexigenic response [71]. Although there is an association between cytokine production and specific gut microbiota in a healthy population [72], more studies are needed in ED patients. 

Morkl et al., found that athletes displayed the most diverse gut microbiota, while obese participants and AN patients displayed less diversity [69]. Nevertheless, Mack et al., did not find differences in microbiota diversity between AN patients and normal weight (NW) controls, probably because the high fiber intake of AN patients may have protected against the estimated reduction of alpha diversity [66]. Moreover, impaired microbiota, SCFA profiles, and gastrointestinal complaints remained persistent after weight gain, whereas overall species richness increased [66]. Significant differences in the composition of intestinal microbiota were found in patients with AN during renourishment. The *Ruminococcaceae*, a family associated with bowel inflammation, were prevalent in AN patients [73].

Distinct alterations in microbiota were observed for individuals with restrictive and binge/purging AN-subtypes. During weight gain, microbial richness increased; however, perturbations in intestinal microbiota and SCFA profiles in addition to several gastrointestinal symptoms did not recover [66].

A recent report suggested that *Enterobacteriaceae*, in particular *Escherichia coli* species, can produce an anorexigenic and anxiogenic protein, the caseinolyitic protease b (ClpB), which may impair αMSH involved in satiety and anxiety signaling. Consequently, the increased abundance of Gram negative bacteria might be linked to a higher production of neuropeptide ClpB, which could be a mediator with the gut–brain axis in AN subjects [63].

Borgo et al., demonstrated that the most suitable predictor for intestinal dysbiosis and metabolic changes was the body mass index (BMI) [63]. In contrast, in a case-series study with three AN patients, no associations between the composition of intestinal microbiota and BMI were observed despite significant weight gain during the treatment [74]

**Table 1:** Recent studies focused on the role of microbiota in anorexia nervosa.

Author (Year)	Aim of Study	Type of Study/Population	Methods	Primary Outcomes	Conclusions	Quality of Evidence
Morita 2015 Japan [62]	To compare the fecal microbiota of female patients with AN with those of age-matched healthy female controls	Cross-sectionalFemale patients with AN (*n* = 25), including restrictive (ANR, *n* = 14) and binge-eating (ANBP, *n* = 11) subtypes, compared with age-matched healthy female controls (*n* = 21)	Using the Yakult Intestinal Flora-SCAN based on 16S or 23S rRNA–targeted RT–quantitative PCR technology	- AN patients had: lower amounts of total bacteria and obligate anaerobes including *Clostridium coccoides* group, *Clostridium leptum* subgroup, and *Bacteroides fragilis* group; lower numbers of *Streptococcus* - In the analysis based on AN subtypes, the counts of the *Bacteroides fragilis* group in the ANR and ANBP groups and the counts of the *Clostridium coccoides* group in the ANR group were lower than those in the control group. - The detection rate of the *Lactobacillus plantarum* subgroup was significantly lower in the AN group - The AN group had lower acetic and propionic acid concentrations in the feces - The subtype analysis showed that the fecal concentrations of acetic acid were lower in the ANR group than in the control group	The analysis confirmed a clear difference in the bacterial components between the AN patients and healthy women. Collectively, these results clearly indicate the existence of dysbiosis in the gut of AN patients.	++
Borgo 2017 Italy [63]	To elucidate the possible relationship between nutritional status, and the microbiota-gut–brain axis in AN	Prospective Case-control 15 AN women 15 age-, sex-, and ethnicity-matched healthy controls	Collection of stool sample, dietary evaluation with a three-day food record, psychopathology assessment.	- AN diet: significant lower energy intake, but macronutrient analysis highlighted a restriction only in fats and carbohydrates consumption. - AN intestinal microbiota showed a significant increase of *Enterobacteriaceae*, and of the archeon *Methanobrevibacter smithii* compared with healthy controls. - In contrast, the genera *Roseburia*, *Ruminococcus,* and *Clostridium* were depleted, in line with the observed reduction in AN of total short chain fatty acids, butyrate, and propionate. - Butyrate concentrations inversely correlated with anxiety levels, whereas propionate directly correlated with insulin levels and with the relative abundance of *Roseburia inulinivorans*, a known propionate producer. - BMI represented the best predictive value for gut dysbiosis and metabolic alterations	The gut dysbiosis could take part in the AN neurobiology, in particular in sustaining the persistence of alterations that eventually result in relapses after renourishment and psychological therapy, but causality still needs to be proven.	++
Mack 2016 Germany [66]	To explore if the intestinal microbiota of AN patients is perturbed in comparison to NW participants and whether these perturbations are recovered after weight gain and/or normalization of eating behavior.	Prospective Case-control AN patients before (*n* = 55) and after weight gain (*n* = 44). Control group: normal-weight participants (NW, *n* = 55)	Authors investigated the fecal microbiota and SCFA in these patients before (*n* = 55) and after weight gain (*n* = 44) in comparison to normal-weight participants (NW, *n* = 55) along with dietary intake and gastrointestinal complaints.	AN patients: - Higher levels of mucin-degraders and members of *Clostridium* clusters I, XI, and XVIII and reduced levels of the butyrate-producing *Roseburia* spp. - Elevated branched-chain fatty acid concentrations, being markers for protein fermentation. - Distinct perturbations in microbial community compositions were observed for individual restrictive and binge/purging AN-subtypes. - Upon weight gain, microbial richness increased; however, perturbations in intestinal microbiota and SCFA profiles in addition to several gastrointestinal symptoms did not recover.	The authors showed profound microbial perturbations in AN patients as compared to NW participants These insights provide new leads to modulate the intestinal microbiota in order to improve the outcomes of the standard therapy.	++
Morkl 2017 Austria [69]	To investigate the gut microbiota composition of a large female cohort including different BMI groups and activity levels along with body composition parameters	Cross-sectional study of 106 female participants: AN patients (*n* = 18), athletes (*n* = 20), normal weight (*n* = 26), overweight (*n* = 22), and obese women (*n* = 20)	DNA was extracted from stool samples and subjected to 16S rRNA gene analysis. QIIME was used to analyze data. Anthropometric assessments, ultrasound, bioimpedance analysis, administered depression inventories, laboratory parameters, and dietary intakes	Alpha diversity was particularly lower in AN patients and obese participants compared to other groups, while athletes showed highest alpha diversity. Several categories significantly associated with community structure were identified: body fat parameters, serum lipids, CRP, depression scales, and smoking. Comparative analysis revealed *Coriobacteriaceae* as the only enriched phylotype in AN compared to other entities (LDA score >3.5)	This study provides further evidence of intestinal dysbiosis in AN and sheds light on characteristics of the gut microbiome in different BMI and physical activity groups. These insights point to new modulation possibilities of the gut microbiota that could improve the standard therapy of AN	++
Armougom 2009 France [64]	To assess the relative abundance of *Lactobacillus*, *Methanobrevibacter smithii*, *Bacteroidetes*, and *Firmicutes* divisions in the microbiota of obese subjects, lean subjects, and AN patients using a real-time PCR assay.	Case-control study20 obese subjects, nine patients with AN, 20 normal-weight healthy controls.Age range: 19–36 years	Authors developed an efficient and robust real-time PCR tool that includes a plasmid-based internal control and allows for quantification of the bacterial divisions *Bacteroidetes*, *Firmicutes*, and *Lactobacillus* as well as the methanogen *M. smithii*.	- Reduction in the *Bacteroidetes* community in obese patients (*p* < 0.01). - Significantly higher *Lactobacillus* species concentration in obese patients than in lean controls (*p* = 0.0197) or anorexic patients (*p* = 0.0332). - *M. smithii* was higher in anorexic patients than in the lean population (*p* = 0.0171)	*Lactobacillus* species are linked to obesity in humans.Increase of *M. smithii* in anorexic patients. This increase might represent an adaptive use of nutrients in this population.	++
Kleiman 2017 USA [74]	To characterize daily changes in the intestinal microbiota in three acutely ill patients with AN over the entire course of hospital-based renourishment	*n* = 3 AN patients No controls	Fecal samples were collected on a daily basis from all participants. All samples were collected by unit nurses and nursing assistants trained in collection protocols.	- Significant changes in composition and diversity of the intestinal microbiota over time at the phylum (*n* = 4), class (*n* = 8), order (*n* = 14), family (*n* = 28), and genus (*n* = 68) levels. - REE increased during treatment, in parallel with energy intake and BMI. - REE was not related to composition or diversity of gut microbiota. - Diet- induced thermogenesis reached a peak after 2–3 weeks of treatment	This preliminary case series suggests that even in a state of pathology, individual microbial signatures persist in accounting for the majority of intestinal microbial variation.	+
Pfleider 2013 France [58]	To study for the first time an anorexia nervosa stool sample by culturomics	AN female single patient (21 years)	The stool sample was collected on her first day of hospitalization, before the introduction of tube feeding. The dietary habits of the patient were surveyed	Nineteen bacterial species never isolated from the human gut before were found, including 11 new bacterial species for which the genome has been sequenced, *Firmicutes*, *Bacterioides,* and *Actinobacteria*	This study revealed new bacterial species participating significantly to the extension of the gut microbiota repertoire, which is the first step before being able to connect the bacterial composition with the geographic or clinical status.	+
Gouba 2014 France [59]	The diversity of microeukaryotes in the gut microbiota of an anorexic patient was investigated using molecular and culture approaches	A 21-year-old Caucasian woman was admitted in an intensive care unit for severe malnutrition in AN	One stool specimen was collected from the anorexic patient	Culture and PCR-based explorations yielded a restricted diversity of fungi but four microeukaryotes, *Tetratrichomonas* sp., *Aspergillus ruber*, *Penicillium solitum,* and *Cladosporium bruhnei,* previously undescribed in the human gut.	Establishing microeukaryote repertoire in gut microbiota contributes to the understanding of its role in human health.	+
Hanachi 2018 France [67]	Authors aimed to determine an association between FIDs severity and dysbiosis of the gut microbiota in a severely malnourished patients with AN undergoing enteral nutrition.	33 AN patients (BMI: 11.7 ± 1.5; Age: 32 ± 12) and 22 healthy controls (BMI: 21 ± 2; age: 36 ± 12)	Fecal microbiota of AN (DSM IVr criteria) female inpatients were collected and compared to healthy controls based on 16S rRNA profiling. The severity of FIDs was evaluated in patients and healthy controls using Francis Score.	- Some potentially pathogenic bacterial genera (*Klebsiella*, *Salmonella*) were more abundant in AN patients, whereas bacterial genera *Eubacterium* and *Roseburia* involved in immune balance were significantly less abundant in patients than controls. - Severity of FIDs was strongly correlated with several microbial genera (r = −0.581 for an unknown genus belonging to *Peptostreptococcaceae* family; r = 0.392 for *Dialister*; r = 0.444 for *Robinsoniella;* and r = 0.488 for *Enterococcus*). Other associations between dysbiosis, clinical, and biological characteristics were identified including severity of undernutrition.	-A marked dysbiosis was identified in AN patients compared to healthy controls. -Observed gut microbiota dysbiosis in malnourished patients with AN is correlated with the severity of FIDs and other metabolic disturbances, which strongly suggests an altered host–microbe symbiosis.	+
Prochazkova 2019 Czech Republic [75]	The change in the gut microbiome and microbial metabolites in a patient suffering from severe and enduring AN and diagnosed with SIBO was investigated.	FMT in a single AN patients	This study assessed the effects of FMT on gut barrier function, microbiota composition, and the levels of bacterial metabolic products.	- Very low bacterial alpha diversity, a lack of beneficial bacteria, together with a great abundance of fungal species were observed in the patient stool sample before FMT. - After FMT, both bacterial species richness and gut microbiome evenness increased in the patient, while the fungal alpha diversity decreased. The total SCFA levels gradually increased after FMT. Contrarily, one of the most abundant intestinal neurotransmitters, serotonin, tended to decrease throughout the observation period	The patient treatment with FMT led to the improvement of gut barrier function, which was altered prior to FMT	+
De Clercq 2019 The Netherlands [76]	To describe FMT in a single patient with AN	26-year-old female following clinical recovery from AN (restricting type)	FMT was performed with feces from an unrelated healthy female donor with a BMI of 25. Dietary intake was reported through online application seven days prior to each visit. Changes in metabolic parameters and body composition were assessed at baseline, six, 12, and 36 weeks.	- The patient gained 6.3 kg in bodyweight (from 45.8 to 52.1 kg), mostly due to a 55% increase in body fat and despite a reported stable caloric intake. Resting energy expenditure was decreased on all post-measurements compared to baseline. - Gut microbial composition showed an increase in weighted phylogenetic diversity at six and 12 weeks with an especially marked increase in the number of *Verrucomicrobia*. -The gut microbiota composition slowly changed back towards the patients’ initial personal core microbial composition. No side effects from FMT were reported or observed during the entire study period.	Authors showed for the first time that FMT induced weight gain in a patient with recurrent AN, suggesting that gut dysbiosis may be one of the causal factors in the etiology of persistent underweight in AN.	+

Abbreviations. AN: anorexia nervosa; ANBP: anorexia nervosa binge eating/purging type; ANR: anorexia nervosa restricting type; BMI: body mass index; CRP: C-Reactive Protein; DSM: diagnosis and statistical manual of mental disorders; FIDs: functional intestinal disorders; FMT: fecal microbiota transplantation; LDA: linear discriminant analysis; NW: normal weight; QUIME: quantitative insights into microbial ecology; REE: resting energy expenditure; SIBO: small intestinal bacterial overgrowth; Quality of evidence grades: low (++), very low (+).

### 2.3. Microbiota Involvement in Bulimia Nervosa and Binge Eating Disorder

BN is characterized by recurrent episodes of binge eating and compensatory behaviors such as self-induced vomiting, laxative or diuretic abuse, fasting, or intensive exercise designed to undo or compensate for the effects of binge eating [77]. Binge-eating disorder shares some characteristics with BN: recurrent episodes of eating large quantities of food; a feeling of a loss of control during the binge; experiencing shame, distress, or guilt afterwards; and not regularly using unhealthy compensatory measures to counter the binge eating [77]. The etiopathogenesis of both disorders is poorly understood. Genetic factors [78,79,80], neurotransmitters, and neurohormonal peptide secretion disturbances [80,81] have been involved in BN and BED. Recently, the gut microbiota has been considered a modulator of host metabolome, inflammation processes, and brain function [80]. Despite gut microbiota in EDs has acquired increased interest in recent years, there are scarce studies on the microbiota in BN and BEDs.

The gut microbiota of BED obese patients has a specific composition and differs from that of obese subjects without BED. A cross-sectional study of a 101-patient cohort using the microbial 16S rDNA sequencing showed decreased *Akkermansia* and *Intestinimonas*, and elevated *Bifidobacterium*, *Roseburia,* and *Anaerostipes* in BED obese patients (Table 1) [82]. Changes in the profile of gut microbiota entail biological consequences, sometimes related to the eating behavior. *Akkermansia muniphila* produces SCFAs (propionate, an important regulator of satiety) and acetate and increases the intestinal levels of several acylglycerols (2-OG, 2-arachidonylglycerol, and 2-palmitoyl glycerol) involved in the regulation of the inflammation and immunity reactions. Therefore, *Akkermansia* has an impact on food intake behavior through the modulation of gut peptides [83,84]. Moreover, this genus is associated with improved insulin-resistance and obesity. Thus, the decreased *Akkermansia* observed in BED obese patients may be harmful. *Bifidobacterium* and *Roseburia* are related to cardiometabolic benefits, such as the reduction of hypertension and atherogenesis [82]. *Anaerostipes* is suggested to regulate human behavior. This genus is increased in psychiatric disorders such as depression and bulimia nervosa. *Intestinimonas* can metabolize toxic products from processed foods, such as Amadori products. This bacteria can convert lysine into butyrate and acetate, involved in the maintenance of a proper gut function [85]. Therefore, the decrease observed in *Intestinimonas* in BED patients may be negative. Hence, gut microbiota may be a modulator factor of metabolic profile of obese BED patients.

As mentioned previously, one of the molecular pathways involved in the regulation of anxiety and satiety is mediated by α-MSH. The caseinolytic protease B produced by *Escherichia coli* is a conformational antigen-mimetic protein of α-MSH with anorexigenic effect. Plasma levels of ClpB depend on the ClpB concentration in gut microbiota [86]. Preclinical models in Wistar rats showed sex-related different response to *E. coli* feeding. Females had *E. coli* in gut microbiota before the intervention, but not males. After *E. coli* feeding, males presented an increased production of α -MSH Ig M compared to females. However, females respond to the intervention by producing higher α-MSH Ig G levels. Females also presented a higher weight gain associated with Ig G and more efficient stimulation of α-MSH. Furthermore, food restriction was associated with ClpB production in the gut microbiota [87]. ED patients have elevated ClpB concentrations in plasma compared to healthy population. ClpB plasma concentrations in the three subgroups of ED patients (AN, BN, and BED) were associated with α-MSH-reactive Ig G, but not statistically significant differences were found when compared the ClpB plasma levels in the subgroups of ED patients. In BED patients, the ClpB concentration correlated with disorder duration, but no association was found with the frequency of binge-eating episodes in BN and BED patients. ClpB plasma concentrations also have been associated with psychopathological traits of these patients [86]. These data support the relation between the *E.coli* ClpB production and ED diagnosis and sex-related differences.

Different factors are involved in the etiopathogenesis of BN and BED, but there is a lack of studies offering a global analysis of the etiopathogenic factors of these disorders. The Binge Eating Genetics Initiative (BEGIN) arises with the aim of expanding the knowledge about the etiopathogenesis of BN and BED. This study will include 1000 patients diagnosed with BN and BED in order to characterize the disease through the collection of saliva, feces, and the register of behavior traits. The study of the genome, the gut microbiota, and the behavioral factors of this cohort will allow exploring the etiology, risk factors, natural history, and response to treatment of patients diagnosed with BN and BED [88]. The phenotyping of these patients will allow in the future the offering of personalized therapeutic options, in many cases related to gut microbiota (Table 2).

### 2.4. Therapeutic Tools in Anxiety and Eating Disorders

Although current pharmacological treatments for anxiety disorders are safer than a few decades ago, the effectiveness in some of them has not improved and can generate addiction problems. Benzodiazepines (BZs) or serotonergic anti-depressants (ADs) are the most widespread therapeutic options, BZs being more efficacious than ADs for reducing GAD symptoms [91]. However, BZs are not recommended for patients with a history of drug abuse, nor can it be prescribed indefinitely. Moreover, BZs are related to some side effects, and high doses may be associated with dementia [92]. Therefore, the development of new tools to look after or restore mental health without the undesired effects discussed above are necessary. A psychobiotic is a live organism that, when ingested in adequate amounts, produces a health benefit in patients suffering from psychiatric illness [93]. The growing preference for preventive medicine coupled with a growing portfolio of products and a greater understanding of their mechanism of action will make psychobiotics a key player in the area of nutritional supplements.

Preclinical studies show that probiotics can alter the cognitive and emotional processes modulating behaviors and brain processes via the gut–brain axis in animal models, such as zebrafish, mice, or piglets [94,95,96]. As previously stated, anxiety is significantly more frequent in subjects with EDs than the general community and may predispose subjects to developing AN or BN [94,97]. For this reason, it was necessary to review all the clinical studies to date of the use of probiotics or postbiotics in patients that suffered anxiety or worked in stressful environments. For that, those studies in which the population had some disease concomitant (irritable bowel syndrome, major depression, autism, autoimmune disorders, etc.), were discarded. 

A total of 14 studies published until 2020 were included in the analysis: twelve of them referred to a probiotic treatment, while the remaining two used a postbiotic [98] or a symbiotic [99] (Table 3). Regarding the duration of the treatments, these varied from 28 days [100] to six months [98], three months being the most used duration. In reference to probiotic intake, only three studies with the same strain (*Lactobacillus casei* Shirota YIT9029, LcS) in fourth grade medical students in exam period did not detect a significant decrease in anxiety according to the State–Trait Anxiety Inventory (STAI) or the Hospital Anxiety and Depression Scale-Anxiety (HADS-A) scores compared to placebo [101,102,103]. Neither did it improve depression rates according to the Hospital Anxiety and Depression Scale-Depression (HASD-D) and Zung Self-Rating Depression Scale (SDS) or decreased salivary cortisol [102]. However, in their last study, they observed a significant positive effect of LcS treatment on sleep scores related to sleepiness on rising and increased sleep length. In addition, overnight single-channel electroencephalography (EEG) recordings showed that LcS strain suppressed sleep latency and increased sleep intensity [101]. The symbiotic mixture composed of 10 g of resistant maize starch and nine probiotic strains also failed to significantly reduce anxiety levels according to the HADS-A scale after six weeks of treatment. Nonetheless, the authors observed a relevant improvement in these female healthcare workers, since after probiotic supplementation all anxiety, depression, and fatigue scores fell into normal ranges [99]. In all studies identified, only one used a single strain of *Bifidobacterium* [104]; by contrast, five studies used a mixture of probiotics strains [99,100,105,106,107] and eight studies used *Lactobacillus*, being *L*. *plantarum* and *L. rhamnosus* the most used species [98,101,102,103,108,109,110,111]. *Bifidobacterium longum* 1714 strain was evaluated under a cold-pressor test in 22 healthy volunteers and after a month of treatment, the anxiety score (STAI) did not significantly increase under the stressor, in contrast to placebo group [104]. This finding is important, since the study was carried out with individuals who did not have baseline anxiety, so these results open the door to the use of probiotics for the prevention of anxiety states. Although more studies would be needed with other different stressors to corroborate this preventive effect. The longest treatment (six months) corresponded to the only study that was done with postbiotics (heat treated *Lactobacillus gasseri* CP2305). In this randomized double-blind placebo controlled trial with medical students under final examination pressure, authors showed a significantly reduction of State Trait Anxiety Inventory (STAI)-trait scores after postbiotic treatment compared to placebo (−1.9 vs. +1.1). Students also increased significantly their sleep quality (according to Pittsburg Sleep Quality Index (PSQI) score) and showed lower depression scores using the General Health Questionnaire (GHQ-28), although there was no significant difference in the global GHQ-28 scores between groups. The probiotic group also showed a non-significant improvement of anxiety and depressive moods (HADS) [98]. This study, moreover, was the only one among the 14 selected that analyzed the change in the microbiota after treatment. Results showed that stress in the students significantly decreased *Bifidobacterium* and increased *Streptococcus* in placebo group. By contrast, the heat treated probiotic significantly mitigated the reduction in *Bifidobacterium* and prevented the elevation of *Streptococcus*. These promising results call for the need to increase the number of clinical studies with postbiotics for the prevention and treatment of anxiety.

Recently, Ma et al., (2021) analyzed, in a follow-up work, the gut microbiota from the study of Lew et al., (2019) to elucidate the mechanism behind the clinical efficacy of *L. plantarum* P8 in significantly reducing some stress and anxiety symptoms [112]. Like the *L. plantarum* DR7 strain, P8 strain significantly decreased TNF-α and interferon-gamma (IFN-γ) after treatment. However, DR7 strain also significantly decreased plasma cortisol levels, in addition to enhancing the serotonin pathway and increasing IL-10 levels [109]. The analysis of the fecal metagenomes from the study with *L. plantarum* P8 showed a significant decrease of the Shannon diversity index in the placebo group but not the probiotic group after 12 weeks. Moreover, the prevalence of some species-level genome bins (SGBs) related to neuroprotective properties significantly increased (e.g., *B. adolescentis*, *B. longum,* and *F. prausnitzii*) after the treatment with *L. plantarum* P8 [112].

The interactions between eating behavior and microbiome modulate host biology. The microbiota has the ability to modulate HPA axis, behavior, neuronal, and immune system. Thus, manipulation of the gut microbiota may be useful to alter the natural history of the EDs.

Increased use of antibiotics in patients with BN and BED prior to the onset of the ED indicates the existing dysbiosis in these patients [89]. Intestinal microbiota in ED patients are different from the healthy population. Microbial gut composition of AN patients shows specific characteristics; butyrate-producers (such as *Roseburia* spp.) are decreased and mucin-degrading bacteria (for example, *Akkermansia muciniphila* and *Methanobrevilacter smithii*) are increased compared to controls [20]. Moreover, the study of intestinal microbiota in the obese population found a different pattern of intestinal microbiota in patients diagnosed with BED (decreased *Akkermansia*, *Desulfovibrio,* and *Intestimonas*, and increased *Anaerostipes*) [82]. Additionally, ClpB produced by *Escherichia coli* (a conformational antigen-mimetic protein of α-MSH with anorexigenic effect) is present in human plasma of patients with EDs [86]. On the other hand, studies in specific populations with malnutrition showed a nutritional recovery of the subgroup treated with antibiotics [113]. Therefore, antibiotics may be a therapeutic target for the existing dysbiosis in patients with EDs. However, researchers have to be cautious in using this therapeutic strategy, because antibiotics can also produce intestinal dysbiosis. More studies are necessary to clarify the effect of antimicrobial therapy on the course of the EDs. Future research should address the search for therapeutic target molecules for the eradication of the desired bacteria.

There is a growing interest in the immunomodulatory role of prebiotics and probiotics for the treatment of mood disorders. A recent meta-analysis of 34 controlled clinical trials evaluating the effects of prebiotics (all with bifidogenic properties) and probiotics (mostly lactobacilli and *Bifidobacterium*) on depression and anxiety concluded that prebiotics have no effect on psychologic disorders, while probiotics have antidepressant and anxiolytic effects [114]. The association of anxiety with EDs is accepted. Probiotic supplementation with lactobacilli and bifidobacteria, by stimulating a cross-feeding mechanism and increasing *Roseburia* abundance and butyrate production, would ameliorate the imbalance of gut structure in AN patients [115]. Future research on this field might offer a new alternative in the therapy of these disorders.

Finally, FMT is being evaluated in the management of the EDs. Preclinical studies in a mice model showed the positive effects in the reverse of compulsive behavior of germ-free mice previously reconstituted with the microbiota of restricting-type of AN patients [38]. Similarly, weight gain was achieved in an AN patient with recurrent underweight after FMT (*Firmicutes*, *Bacteroidetes*, *Verrucomicrobia,* and *Euryarchaeota*) [76], while the other case report showed no clinical improvement after FMT (*Akkermansia muciniphila*, *Methanobrevibacter smithii*) [75]. The total SCFA were increased [75,76] and serotonin decreased after FMT [75]. On the other hand, *Firmicutes*, *Rikenellaceae*, *Ruminococcaceae*, *Clostridiaceae,* and *Prevotellaceae* are families of bacteria negatively associated with weight gain and positively associated with gut microbiota ClpB KEGG function (K03695, Kyoto Encyclopedia of Genes and Genomes annotation) after fecal transplantation from humans to mice [116]. Therefore, future studies to examine the role of fecal microbiota transplantation in eating behavior and weight gain may be helpful in unraveling altered pathways in EDs.

## 3. Discussion

The composition of the gut microbiota is strongly modulated according to the characteristics of the diet [50,117], Moreover, the gut microbiota plays a critical role in weight gain and energy intake from the aliments. For that, disruptions in the balance of human microbiota are associated with several diseases, such as diet-related mental illness [118] or generalized anxiety disorders. Therefore the scientific research focused on the gut microbiota profile in patients diagnosed with anxiety or ED leads to an innovative approach to understanding the etiopathogenesis of EDs and anxiety.

Several studies show imbalances in the intestinal microbiota of patients with EDs and anxiety. In GAD, for example, there is an increase of bacterial groups with inflammatory capacity such as *Ruminococcus gnavus, Fusobacteria,* or *Escherichia*–*Shigella*, positively associated with anxiety severity and a lower prevalence of SCFA-producing genera, whose lack triggers an intestinal barrier dysfunction [119]. Therefore, an increase of pathogenic bacteria able to degrade gastrointestinal mucins and produce exotoxins and inflammation could exacerbate anxious symptoms. In contrast, *Prevotella* correlated positively with anxiety reduction [47]. This is particularly interesting, because a significantly tighter connection between emotional well-being and a *Prevotella*-dominant condition [120] as well as an increased response to affective images in the limbic system has been suggested [121]. *Faecalibacterium,* related with anxiolytic and antidepressant-like effects, was also decreased in GAD population. *F. prausnitzii* increase SCFAs and IL-10 levels and reduce corticosterone and IL-6 levels in rats under a chronic unpredictable mild stress [122]. Therefore, their depletion can increase the anxiety states. On the other hand, the increase of *Enterobacteriaceae*, together with a weakened intestinal barrier, would enhance the translocation of the proinflammatory endotoxin LPS in blood [123]. LPS produces an inflammatory cascade that activates the kyneurine pathway; tryptophan will be degraded by indoleamine 2,3-dioxygenase (IDO), decreasing the serotonin levels [124].

Although it has not been clarified if gender influences the shaping of the microbial community in patients with GAD, if it seems that the medication does [42]. However, this exogenous factor has not been adequately considered by studies. Of the three studies comparing the microbiota of healthy controls with GAD patients, only Jiang et al., (2018) examined a subgroup with treatment-naïve patients. Furthermore, in six of the 14 studies with probiotics or postbiotics, no data on the use or not of psychotropic drugs during follow-up were reported [99,100,105,106,108,109]. Psychotropic medication is a significant source of inter-study variation, since, in addition to change the microbial community, its absorption and efficacy can be affected by the patient′s own microbiota [125,126]. Therefore, this variable must be taken into consideration in future studies where the main aim is the reduction of symptoms in these patients through microbiome modulating solutions.

During this review, we have found numerous studies and reviews with microbiota modulators that tested the changes in anxiety levels. However, studies generally focused on patients with other pathologies, especially individuals with irritable bowel syndrome and major depression, as other authors also point out [114]. For this reason, as well as for the co-occurrence with EDs, we considered it necessary to collect those studies focused on healthy individuals with anxiety or those subjected to stressful conditions that can lead to anxious states. 

One of the main conclusions of the present study is that it seems that the effect of probiotics in reducing anxiety seems all the more effective the higher the baseline anxiety level of the individual. Of the 14 studies, seven reported basal anxiety or stress based on the HADS-A [99], STAI [101,103,107], Hamilton Rating Scale for Anxiety-A (HAM-A), [106] or Perceived Stress Scale 10 (PSS-10) scales [108,109]. Of these seven studies, five achieved a positive outcome in reducing anxiety, despite using different treatments and conditions [99,106,107,108,109], while the same strain (*L. casei* Shirota) failed to decrease subjective anxiety levels in the other two remaining studies. This observation coincides with the sub-analysis of two groups with different baseline levels of distress, where no significant improvement was observed in the normative distress group after probiotic treatment [100]. Similarly, this occurs in depressive symptoms, where a meta-analysis with a total of 1349 patients showed that probiotic supplementation significantly improved the moods of individuals with mild–moderate depressive symptoms versus healthy individuals [127]. This trend is also observed in mice, but not rats, according to a meta-analysis, where a subgroup analysis revealed that probiotic administration significantly reduced anxiety-like behavior in diseased but not in naïve animals [94].

The studies in this review bring up some points that deserve to be considered for future studies in the anxiety field. First, we have not found a relationship between the main outcomes and the use of a multi strain or a single strain product. This fact corroborates once again that it is the intrinsic characteristics of the strains and their combinations and not the number of strains added in a product that determine its efficacy. A recent review based on 65 randomized clinical trials (RCTs) for eight different diseases remarks on our findings and shows how, in most cases, multi-strain mixtures are not significantly more effective than single-strain probiotics [128]. Secondly, we found a great diversity of questionnaires used to measure anxiety levels, questionnaires based on self-reports and not supervised by specialized personnel. In one study, authors used modified questionnaires of STAI6 and Edinburgh Postnatal Depression Scale (EPDS), which was not validated [110]. This makes it difficult to obtain comparable results among studies. For this reason, the use of specific biomarkers and the choice of the right questionnaire for the right population is needed. One should be cautious about the title of the instrument and find a compromise between precision and respondent burden [129]. Finally, it is necessary to mention the few studies with postbiotics in the field, only one, despite the growing interest that this “-biotic” generates due its several advantages over probiotics, in terms of stability or use in several food matrices.

Interestingly, two studies measured plasma pro-inflammatory cytokines such as IFN-γ and TNF-α after probiotic treatment, obtaining reduced levels as anxiety decreased. Moreover, these two cytokines were significantly correlated with psychological traits of Depression Anxiety Stress Scale 42 (DASS-42) (anxiety and stress), indicating that inflammation is probably a major cause of stress and anxiety [108,109]. In fact, inflammation affects anxiety-related brain regions such as amygdala, insula, and anterior cingulate cortex [130]. Therefore, the use of specific probiotic strains with anti-inflammatory effects could be a powerful coadjuvant tool in the treatment or prevention of anxiety, which, as mentioned, is usually linked to other pathologies, such as EDs.

In addition to their anti-inflammatory effects, probiotics can also act on anxiety states in various ways, although we insist again that these characteristics will vary depending on the probiotic strain chosen. First, probiotics can attenuate the increased and prolonged activation of the HPA axis in GAD patients. In murine models, *B. breve* CCFM1025 significantly reduced the hyperactive HPA response, possibly via regulating the expression of glucocorticoid receptors (*Nr3c1*) [131]. These results are remarkable, because persistent HPA hyperactivity has been associated with higher rates of relapse, and glucocorticoid receptor function is impaired in anxiety disorders [132]. Other probiotic strains can also regulate the HPA axis, improving the systemic and nervous antioxidant status [133], reducing cFos protein expression in different brain areas, and changing the brain plasticity due to BDNF production [134]. Second, some strains from *Bifidobacterium* genus such as *B. adolescentis* PRL2019 and *B. adolescentis* HD17T2H can stimulate the in vivo production of GABA [44]. This inhibitory neurotransmitter and its neurotransmitter system is the target of BZs to treat anxiety disorder [135]. In zebrafish, probiotics can also modulate the GABAergic pathway through the differential expression of genes such as *gabra1* that encode the GABA-A alpha 1 receptor and serotonin transporter A (*slc6a4a*) [136]. Some probiotics can also increase the serotonin pathway and maintain the levels of norepinephrine and dopamine, which are higher in stressed subjects [109]. Finally, oxidative stress and intestinal permeability are also therapeutic targets and fields of enormous interest in probiotics due to their relationship with anxiety [137,138]. The increase of antioxidant enzymatic and non-enzymatic defenses in rat brains generated neuroprotective effects and reduced compulsiveness/anxiety using a marble burying test and open field test [93]. Interestingly, the strain *B.longum* CECT 7347 that showed antioxidant capacity in the nematode *C. elegans* [139] and improved the structure of the intestinal epithelium in a murine model of enteropathy by gliadin [140] also decreased anxiety behaviors in zebrafish [95]. *Weissella paramesenteroides* WpK4 also exerted their beneficial roles in the gut–brain axis through the reinforcement of the intestinal barrier in murine models of colitis and chronic stress [141]. Therefore, the effect of probiotics on anxiety cannot be attributed to a single mechanism of action, nor do all probiotics have the same characteristics to exert their effect on anxiety. Physical and psychological characteristics of AN patients entail differences in their gut microbiota composition [58,59]. Different studies have shown the involvement of the intestinal microbiota in neuroendocrine and metabolic pathways, including SCFA and anorexigenic hormones like αMSH [52], as well its association with gut inflammation. Different microorganisms are associated with the features of the disorder: *Bifidobacterium spp.* and *Lactobacillus spp.* are involved in the regulation of anxiety; *Enterococcus spp., Escherichia spp., Streptococcus spp., Candida spp.* and *Bacillus spp.* are related to mood regulation [53,54]. Furthermore, LPS produced by gram-negative bacteria are related to the regulation of food intake [55]. Although several studies are conducted in order to recognize the dysbiosis of AN subjects, well-designed studies are needed for a complete understanding of the disbalances of gut microbiota of these patients, in addition to more research aimed at knowing the molecular pathways responsible for the disorder.

Despite the studies reporting on the intestinal dysbiosis in AN patients, reports about the composition of gut microbiota of BN and BED patients are scarce. The main molecular pathway studied is the anorexigenic and anxiogenic caseinolyitic protease b (ClpB) produced by *Eschericchia coli* species. Studies in AN patients showed an increased abundance of *E. coli* linked to the production of the ClpB neuropeptide [63]. Although there are no human studies in BN and BED, preclinical models of EDs show the mediator effect of ClpB with the gut–brain axis [51,87,90]. Initiatives such as BEGIN [88] are necessary to understand the gut microbiota composition in these patients.

This review shows some of the desirable characteristics in future microbiome-based solutions for eating disorders: first, the decrease of the anorexigenic ClpB protein in an AN patients, whose plasma concentration is correlated with proportion of *Enterobacteriaceae* in faeces [142]. The screening of probiotic strains with antagonistic properties against ClpB protein producers could be helpful in the treatment of EDs. Second, the modulation of the ghrelinergic signaling to balance the feeding behavior could be another mechanism for the development of new therapeutic tools. In fact, different *Lactobacillus* and *Bifidobacterium* strains are able to modulate the ghrelin receptor (GHS-R1a) [143]. Finally, the increase of butyrate-producing gut commensals by cross-feeding from some species of *Bifidobacterium* could stimulate epithelial cell proliferation. Butyrate would reinforce the intestinal wall, resulting in a larger absorptive surface to improve nutrients absorption from the diet [144]. As is discussed above, anxiety can promote Eds, and they often appear together. Therefore, reducing anxiety levels through microbiome modulators could be an additional strategy to prevent the EDs.

Knowledge of the intestinal microbiota will allow progress in the study of the patophysiological mechanisms of the EDs in order to establish possible therapeutic options as probiotics or fecal microbiota transplantation. The correction of dysbiosis may be associated with the physical and emotional well-being of ED subjects.

## 4. Materials and Methods 

**Figure 1:**
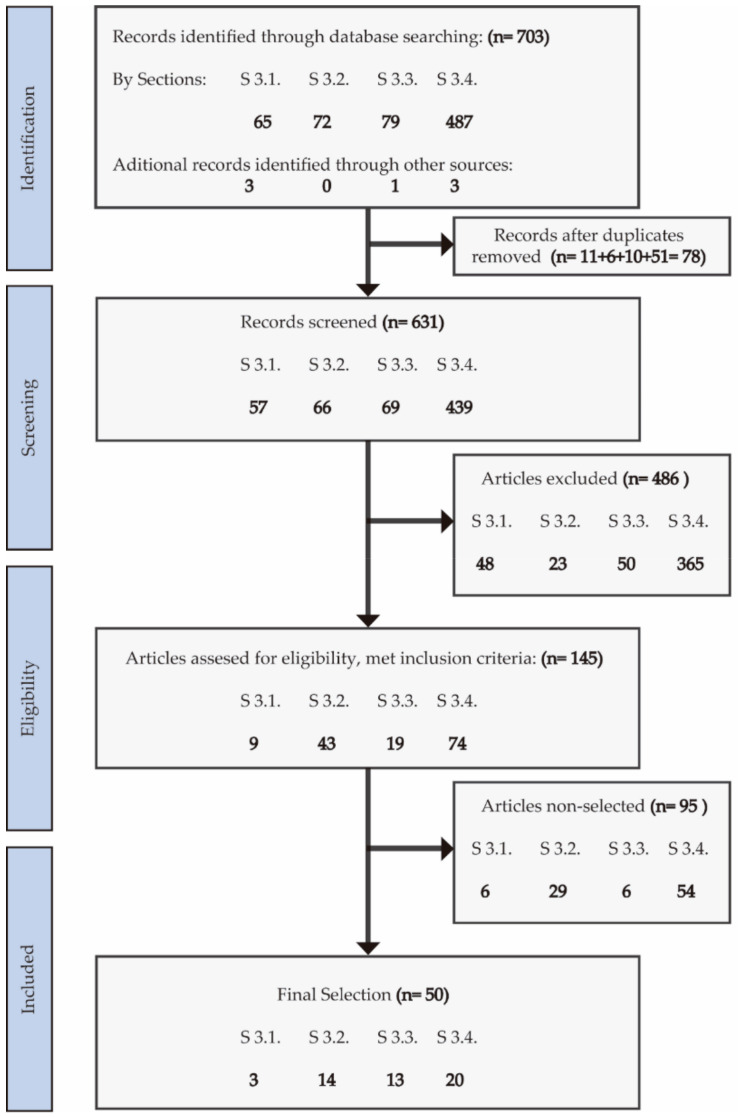
Methodological flowchart following preferred reporting items for systematic review based on PRISMA.

This systematic review has been performed following the PRISMA statement for systematic reviews and meta-analyses [145,146], which includes definition of the research question and bibliographic search; data collection, evaluation, comparison, and synthesis; and critical analysis and findings presentation, indicating the strengths and weakness of the studies evaluated (Figure 1). A meta-analysis to evaluate the role of microbiota in anxiety and/or EDs was not considered due to the experimental design and clinical differences observed among the studies selected as well as by the low sample size achieved in these studies, which would generate an important bias in the statistical results. 

The bibliographic search strategy was conducted to identify all studies reporting microbiota alterations in patients diagnosed with anxiety and/or EDs (AN, BN, or BED), also highlighting the use of probiotics as therapeutic tools. The electronic databases consulted were PubMed (MeSH), Cochrane Central Register of Controlled Trials, and Scopus. The following descriptors were used (as MeSH terms or not) with Boolean operators (AND/OR) in multiple combinations (see Supplemental Material S1): Section 2.1. “((fecal microbiota) OR (gut microbiota)) AND (generalized anxiety disorder)”; Section 2.2. “(microbiota OR microbiome OR dysbiosis) AND (anorexia nervosa)”; Section 2.3. “(microbiota OR gastrointestinal microbiome OR dysbiosis) AND (bulimia nervosa OR binge-eating disorder)”; Section 2.4. “Probiotics AND (anxiety OR anorexia nervosa OR bulimia nervosa OR binge-eating disorder)” and ” (Psychobiotic OR probiotic OR prebiotic OR symbiotic OR fecal transplantation OR postbiotic) AND (generalized anxiety disorder OR anxiety)”.

Inclusion criteria were papers written in English and Spanish (with no geographical restrictions) published from January 1, 2009 to December 31, 2020; the presence of the selected terms in the title or as keywords; original research performed in humans; selected experimental designs including clinical trials, case–control, longitudinal cohort, cross-sectional, and case report studies. Sample size ≥ 10. The quality of controlled studies referring to randomized, nonrandomized, and pre-post treatment was critically appraised following the Cochrane Collaboration’s Risk of Bias Tool [147]. Exclusion criteria were infant population to 10 years old and no association with other psychiatric diseases. Interventions using only prebiotics or immunotherapy were also excluded for Section 2.4.

The selection of original manuscripts started by screening titles and abstracts for inclusion, creating a reference list of relevant papers for the topics explored in this review. Two investigators (T.L. and N.-T.E.) conducted each stage of the studies selection, deleted duplicate inputs, and reviewed studies as excluded or requiring further assessment. All data were extracted by one investigator (T.L.) and cross-checked by a second investigator (N.-T.E.). In case of discrepancies in the selected studies, we opted for reconciliation through team discussion. The information evaluated from each study was first author, experimental design, number of participants, control groups, main outcomes/findings, conclusions, and strengths and limitations (including biases). The eligibility criteria followed the PICOS (patient, intervention, comparators, outcome, and study design) approach. Population: Men or women diagnosed with anxiety or ED (anorexia, bulimia, or binge-eating disorder); intervention: if applicable, any doses, strains, or species of probiotics administered; comparators: if applicable, placebo or no probiotics; outcome: the primary outcome was microbiota composition in anxiety or ED patients. All authors performed a critical appraisal for the studies selected following the inclusion criteria, also analyzing the methodology and key results. 

In the literature evaluated following PRISMA methodology, we observed heterogeneous results due to the different populations (patients with different ED) compared; the distinct health conditions derived to the clinical complexity of ED diagnosis; the reduced number of randomized trials in ED patients using probiotics; and the small sample size observed in these studies. Finally, the studies indicated in Figure 1 for each section of this review were identified through databases searching, selecting after meeting the inclusion criteria, and the application of the exclusion criteria the following: Section 2.1 (3), Section 2.2 (14), Section 2.3 (13), and Section 2.4 (20).

Finally, the articles were evaluated using the GRADE (Grades of Recommendation, Assessment, Development, and Evaluation) approach, which describes four levels of quality of evidence: high, moderate, low, and very [148]. Quality of evidence was obtained (Table 4) after judging the designs of the studies, the risk of bias, inconsistency, indirectness, imprecision, number of subjects, and publication bias.

## Figures and Tables

**Table 2:** Main studies based on microbiota composition in Bulimia nervosa and Binge Eating Disorder and microbiota studies.

Author (Year)	Objective	Type of Study and Sample Size	Inclusion and Exclusion Criteria	Interventions	Outcomes	Conclusion	Quality of Evidence
Raevuori (2016) [89]	To examine the use of antimicrobials as an indicator for infection prior to the onset of the ED.	Case control study. ED patients (*n* = 1592) and controls (*n* = 6368).	IC: patients treated in the Eating Disorder Unit at the Helsinki University Central Hospital form January 2000 to September 2010. Diagnosis of the ED according the ICD-10 criteria. Four controls for each patient matched for age, sex, and place of residence.	Assessment of antimicrobial medication (systemic antibacterial, antifungal, and antiviral therapy) through registries of the Social Insurance Institution of Finland.	BN and BED patients had received more antimicrobial prescription than controls (OR: 1.7, 95% CI: 1.3–2.1 and 2.6, 95% CI: 1.4–4.6, respectively), no differences in AN patients were found. Antibacterial and antifungal therapy. Mean DDD use of antibacterial therapy were increased in BN (*p* < 0.001) and BED (*p* < 0.001) compared to controls. No differences were found in the use of antivirals in any of the EDs. Mean DDD of antifungal medication was increased in BED (*p* < 0.001). Use of antimicrobials was higher in BN and BED (OR: 2.1, 95% CI: 1.6–2.8 and 2.9, 95% CI: 1.6–5.4, respectively) when compared to AN. The use of antibacterial and antifungal was increased in BN and BED compared to AN. The use of antiviral was increased only in BN group compared to AN.	The increased use of antimicrobial therapy in patients with BN and BED indicates a higher number of infections in these groups prior to the onset of the disorder. Infections and antibacterial therapy may contribute to the proxy of the EDs through inflammation and changes in gut microbiota.	+++
Breton (2016) [86]	To verify if ClpB produced by *Escherichia coli* is present in human plasma of patients with ED (AN, BN, and BED).	Case control study. Female patients with AN (*n* = 24), BN (*n* = 29), and BED (*n* = 13) and controls (*n* = 29).	EC: participants with EDs (according to EDI-2) and other psychiatric disorders (according to MADRS)	ClpB immunoassay in plasma	ClpB was detectable in plasma of ED patients and controls. ClpB plasma concentrations were increased in ED patients compared to controls. No statistically significant differences in patients’ subgroups. Positive correlation of ClpB with α-MSH-reactive-IgG in all subgroups of ED patients.	ClpB is present in human plasma. ClpB increased concentration in ED patients supports a link between bacterial ClpB and ED diagnosis.	++
Leyrolle (2020) [82]	To study the fecal microbiome and non-targeted plasma metabolomics of obese BED patients.	Cross-sectional study in obese population (*n* = 101 obese patients) from Food4Gut cohort.	IC: male and female patients, aged 18–65 years, BMI >30 kg/m^2^, Caucasian ethnicity, presence of metabolic obesity-related disorder. EC: use of antibiotics, pre- or probiotics, dietary fibers, or any drug that modifies intestinal transit time within six weeks before the study, pregnancy, heavy psychiatric disorders, use of antipsychotics, current particular diets, excessive alcohol intake, type 1 diabetes, general dislike for vegetables.	Q-EDD Microbial 16S rDNA sequencing Non-targeted metabolomics (liquid chromatography–mass spectrometry)	Subjects with BED showed a decrease in *Akkermansia* (*p* = 0.01), *Desulfovibrio* (*p* = 0.04), and *Intestimonas* (*p* = 0.01), and an increase in *Anaerostipes* (*p* = 0.03). Metabolomics revealed higher level of Bisphenol A (*p* = 0.011) and Isovalerylcarnitine (*p* = 0.006) in BED individuals.	Omics approaches allow the characterization of gut microbiota and plasma metabolites of BED obese subjects.	++
Tennoune (2015) [90]	To compare the effects of *E. coli* on autoantibodies against α-MSH and ACTH according to gender in rats	Preclinical model in Wistar rats (*n* = 48).	Male and female Wistar rats, body weight 220 to 250 g.	Wistar rats received daily *E. coli* K12 in a culture medium by intragastric gavage over a three-week period. Control rats received only a culture medium. Plasma autoantibody assay against α-MSH, ACTH, and ClpB. Locomotor activity and anxiety tests.	*E. coli* was present only in females before gavage. Body weight increase in females and decrease in males after *E. coli* gavage. Plasma levels of anti-α-MSH and ACTH IgG were higher in females independent of the gavage. After *E. coli* gavage, α-MSH IgG was increased in females, and α-MSH IgM in males.	E. coli affects feeding and anti-MSH antibodies in a different way according to gender. Sex-related levels of the different gut bacteria may represent a risk factor for developing an ED.	+
Tennoune (2014) [51]	To study the effect of α-MSH antigen-mimetic protein on α-MSH auto-antibodies production and food intake. To see the association of plasma antibodies of α-MSH levels of patients diagnosed with AN, BN, and BED with EDI-2 score.	Preclinical model and case-control study.	C57Bl6 male mice (*n* = 32). Human subjects (women): controls and cases of AN, BN, and BED disorders (diagnosis according DSM-IV).	*E. coli* K12 culture and protein extraction. Chronic intragastric delivery *of E. coli* in mice. Study of plasma levels of anti-ClpB IgG α-MSH. Locomotor activity and anxiety tests in mice. EDI-2 scores.	Production of anti-ClpB IgG crossreactive with α-MSH influences food intake, body weight, anxiety, and melanocortin receptor 4 signaling in mice. Intragastric gavage of *E. coli* decreased food intake and stimulated formation of ClpB- and α-MSH-reactive antibodies in mice. Patients with AN, bulimia, and BED have increased plasma levels of anti-ClpB IgG crossreactive with α-MSH and correlates with EDI-2 scores.	Bacterial ClpB protein, responsible for the production of auto-Abs crossreactive with α-MSH, is associated with pathologic feeding and emotion in humans diagnosed with EDs.	++
Breton (2020) [87]	To study if ClpB production by enterobacteria can be altered by chronic food restriction and female sex.	Preclinical model in Sprague–Dawley rats (*n* = 24)	12 male and 12 female Sprague–Dawley rats.	Wistar rats received free access to food and water for seven days. Food access was limited during 1.5 h for one week. Plasma collection and feces. ClpB DNA analysis, ClpB, and α-MSH reactive antibody assay. Bacterial culture.	Food restriction increased ClpB levels in feces and plasma in both females and males. Females had higher levels of basal ClpB in plasma and gut and increased levels of ClpB-reactive IgG and IgM. ClpB concentration after the use of estradiol in *E.coli* cultures were lower and testosterone had no effect.	Enterobacterial ClpB antigen may be associated with risk for developing an ED.	+

Abbreviations. ACTH: adrenocorticotropic hormone; AN: anorexia nervosa; BED: binge eating disorder; BMI: body mass index; BN: bulimia nervosa; ClpB: caseinolytic protease B; DDD: defined daily dose; DSM-IV: Diagnostic and Statistical Manual of Mental Disorders, 4th Ed; EC: exclusion criteria; ED: eating disorder; EDI-2: Eating Disorder Inventory-2 scores; IC: inclusion criteria; MADRS: Montgomery–Asberg Depression Rating Scale; MSH: melanocyte-stimulating hormone; Q-EDD: Questionnaire for Eating Disorder Diagnosis. Quality of evidence gradesmoderate (+++), low (++), very low (+).

**Table 3:** Studies focused on the use of probiotics in anxiety disorder.

Author (Year)	Subjects	Design/ Country	Strain (s)/Dose/Duration	Questionnaires/ Techniques Used	Main Outcomes	Conclusions	Quality of Evidence
Tran et al., (2019) [100]	Healthy college students; *n* = 86; 75.6% Hispanic non-white: 27.9% African/African American: 26.7% Caucasian: 23.3%Asian/Pacific Islanders: 16.3%	Randomized DBPC/US	Group A: 18 probiotic species; 5 × 10^10^ CFU/d (*n* = 11) Group B: 10 probiotic species; 5 × 10^10^ CFU/d (*n* = 13) Group C: placebo (*n* = 11) Group D: 18 probiotic species; 15 × 10^9^ CFU/d (*n* = 15) Group E: 10 probiotic species; 1 × 10^10^ CFU/d (*n* = 15)28 d	BAI; ACQ-R; PANAS; NMR;PSWQ	Group A: ↑ positive affect ** and anxiety control *; ↓ in worry * Group B: ↓ in panicanxiety* and negative affect *; ↑ negative mood regulation * Group C: NS changes Group D: ↓ neurophysiological anxiety *Group E: ↓ in negative affect *	Gender and ethnicity might be a covariance to anxiety CFU and species count may not be equally applicable to the probiotics’ effectiveness Species combination, not species count, may be the critical factor in the effectiveness of probiotics Probiotics can have a greater effect on the higher the initial stress level	++
					**By gender:**		
					♀: ↓ in worry * and negative affect ** ♂: ↓ in autonomic anxiety *		
					**By ethnicity:**		
					African/African American: ↓ in negative affect *, neurophysiological anxiety **, subjective anxiety *, panic anxiety *, and BAI total score **		
					**By CFU count:**		
					High CFU (A and B groups): ↓ panic anxiety * and worry *; ↑ positive affect **, and anxiety control * Low CFU (A and B groups): NS changes		
					**By number of strains:**		
					High (A and D groups): NS changes Low (B and E groups): ↓ negative affect **		
					**By distress level at baseline:**		
					High: ↓ BAI *, PSWQ *, PANAS Negative affect *; ↑ ACQ **, ANAS Positive affect *, and NMR * Normative level: NS change		
Smith-Ryan et al., (2019) [99]	Female healthcare workers employed on a rotating-shift schedule; *n* = 41 Caucasian: 93.9% African American: 6%	Randomized DBPC/US	Ecologic^®^ BARRIER (*Bifidobacterium bifidum* W23, *B. lactis* W51, *B. lactis* W52, *Lactobacillus acidophilus* W37, *L. brevis* W63, *L*.*casei* W56, *L. salivarius* W24, and *L. lactis* (W19 and W58); 1 × 10^10^/d + 10g of resistant maize starch; 6 w	HADS; CFQ	HADS-A; HADS-D and CFQ: NS differences between groups Clinically relevant decrease in HADS-A scores (change (Δ): −2.3 ± 2.6) and CFQ-11 scores (Δ: −4.8 ± 5.5) in PG	Potential beneficial effect on anxiety and mental fatigue in a shift-working population. Probiotics can have a greater effect the higher the initial stress level	+++
Nishida et al., (2019) [98]	Medical students in exam period; *n* = 60; 18%	Randomized DBPC/Japan	Heat treated *Lactobacillus gasseri* CP2305; 1 × 10^10^ cells/d); 6 m	STAI; PSQI; HADS; GHQ-28	6 m: significantly reduction of STAI-trait scores in PG compared to CG (−1.9 vs. +1.1) and increase of sleep quality (PSQI) CP2305 ameliorated anxiety and depressive moods vs. placebo (HADS) NS changes in salivary cortisol between groupsSignificant ↓ *Bifidobacterium* ↑ *Streptococcus* in CG vs. PG after 6 m n-Valeric acid significantly increased after postbiotic intake. NS changes in other SCFAs	Long-term use of the probiotic may improve the mental state, sleep quality, and gut microbiota of healthy adults under stressful conditions	+++
Lew et al., (2019) [108]	Stressed adults; *n*= 103; 49.5%	Randomized DBPC/ Malaysia	*Lactobacillus plantarum* P8; 1.2 × 10^10^ CFU/d; 3 m	DASS-42 PSS-10	Total score for stress (PSS-10): NS differences between groups Total score for stress (DASS-42): significant reduction vs. placebo after 4, 8, and 12 w Total score for anxiety (DASS-42): significant reduction vs. placebo at 4 and 12 w. Changes from moderate to normal levels in PG. P8 strain significantly reduced breathlessness **, abnormal heart beats *, and fear * NS effects against reduction of depression and salivary cortisol between groups Significant changes IFN-γ (PG: -0.62 ± 2.98 vs. 7.46 ± 10.88 ug/dL in CG) and TNF-α after treatment (PG: 0.17 ± 1.29 vs. 1.69 ± 1.80 pg/mL in CG)	*L. plantarum* P8 reduces some stress and anxiety symptoms via anti-inflammatory properties and enhances memory and cognitive abilities	++++
Chong et al., (2019) [109]	Stressed adults; *n* = 111	Randomized DBPC/Malaysia	*Lactobacillus plantarum* DR7; 10^9^ CFU/d; 3 m	DASS-42 PSS-10	Total score for stress (PSS-10): NS differences between groups Total score for stress (DASS-42): significant reduction vs. placebo after 8 w in all subjects. After sub analysis, young adults (age <30 years old) showed higher reduction of total DASS-42 stress score compared to young adults in placebo. NS differences for stress score DASS-42 in >30 years old. DR7 strain significantly improved relaxation * and alleviated use of nervous energy * Total score for anxiety (DASS-42): significant reduction vs. placebo after week 8 in all populations studied. DR7 strain significantly improved swallowing ** and reduced trembling ** NS effects against reduction of depression DR7 significantly reduced plasma cortisol levels, IFN-γ, and TNF-α in total subjects compared to the placebo after 12 w. DR7 also significantly increased IL-10 and enhanced the serotonin pathway	*L. plantarum* DR7 reduces symptoms of stress and anxiety, improves cognitive and memory functions, and reduces levels of plasma cortisol and pro-inflammatory cytokines.	++++
Takada et al., (2017) [101]	Fourth-grade medical students in exam period; *n* = 94; 41.4% ♀	Randomized DBPC/Japan	*Lactobacillus casei Shirota* YIT9029 in fermented milk (100 mL); 1 × 10^9^ CFU/mL, 11 w	STAI; OSA/EEG	No difference between groups regarding sleep scores (OSA). After sub analysis, YIT9029 significantly relieved sleepiness on rising and increased sleep length. Initiation and maintenance of sleep, dreaming, and recovery from fatigue did not change YIT9029 significantly suppressed the prolongation of sleep latency and prevented the reduction in N3 sleep No differences in STAI scores between groups	YIT9029 may help to maintain sleep quality during a period of increasing stress.	+++
Slykerman et al., (2017) [110]	Pregnant women in their 14–16 weeks gestation; *n* = 380 Maori: 12.9% Pacific: 2.1% Asian: 7.1% European: 77.6% Other: 0.26%	Randomized DBPC/New Zealand	*Lactobacillus rhamnosus* HN001; 6 × 10^9^ CFU/d; From 35 weeks gestation until six months if breastfeeding	Modified questionnaires of STAI6 and EPDS. No validated	Depression (EPDS): PG reported, retrospectively, significantly lower depression scores (HN001 mean= 7.7 (SD = 5.4), placebo 9.0 (6.0); effect size −1.2, (95% CI −2.3, −0.1), *p* = 0.037) Anxiety (STAI6): PG reported, retrospectively, significantly lower anxiety scores (HN001 mean = 12.0 (SD = 4.0), placebo 13.0 (4.0); effect size −1.0 (−1.9, −0.2), *p* = 0.014)	HN001 may be useful for the prevention or treatment of symptoms of depression and anxiety postpartum.	++
Kelly et al., (2016) [111]	Healthy male volunteers; *n* = 39	Randomized placebo-controlled, cross-over design/Ireland	*Lactobacillus rhamnosus* (JB-1); 1 × 10^9^ CFU/d, 4 w	SECPT; EEG; STAI; BDI; BAI	SECPT: NS differences between groups STAI: NS differences between groups BDI: NS differences between groups BAI: NS differences between groups EEG: NS differences between groups	JB-1 was not superior to placebo in modifying stress-related measures, HPA response, inflammation, or cognitive performance in healthy male participants	+++
Colica et al., (2017) [106]	Healthy subjects; *n* = 33; 83.3% ♀	RCT/Italy	Group A: POS ^#^ + no dietary change (*n* = 11); 1 bag/d Group B: hypocaloric diet; (*n* = 11) Group C: POS ^#^ + hypocaloric diet (*n* = 11) 3 w	HAM-A	Group A: ↓ in HAM-A total score for all study population (*p* = 0.01). Significant reduction in the number of anxious subjects after sub analysis (*p* = 0.03; Δ% = −39.3%) Group B: No differences for all study population Group C: ↓ in HAM-A total score for all study population (*p* = 0.04). All anxious subjects became nonanxious after sub analysis (*p* =0.01; Δ%= −100%)	A balanced diet, associated with this probiotic mixture, has a greater effect on the improvement of anxiety symptoms than probiotic alone	++
Takada et al., (2016) [103]	Fourth-grade medical students in exam period; *n* = 140; 45.7% ♀	Randomized DBPC annually for three consecutive years/Japan	*Lactobacillus casei Shirota* YIT9029 (LcS) in fermented milk (100 mL); 1 × 10^9^ CFU/mL, 8 w	STAI	Significant increase of STAI score in both groups before exam compared to baseline (CG: 37.4 ± 1.1 and 48.7 ± 1.4, *p* < 0.01, PG: 38.7 ±0.8 and 50.7 ±1.2, *p* < 0.01). NS differences between groups NS differences in salivary cortisol levels between groups in each trial. Significant lower salivary cortisol levels in PG before exam when all data were pooled	Daily administration of LcS for eight weeks did not affect subjective anxiety	++++
Mohammadi et al., (2016) [105]	Petrochemical Iranian workers; *n* = 70; 48.5% ♀	Randomized DBPC/Iran	Group A: probiotic yogurt ^§^ + placebo (*n* = 25) Group B: conventional yogurt + probiotic capsule ^°^ (*n* = 25) Group C: conventional yogurt + placebo (*n* = 20) 6 w	DASS-14; GHQ-28	Group A and B: significant improvement of general health after 6 w. (ΔGHQ-28: -4.5 ± 1.7 and -7.1 ± 1.7 in group A and B, respectively) Group A and B: significant improvement of anxiety and depression scores after 6 w. (ΔDASS-14: -10.3 ± 3.9 and -9.5 ± 4.3 in group A and B, respectively) NS changes in kynurenine, tryptophan, neuropeptide Y, cortisol or ACTH levels between groups. No influence on hypothalamic–pituitary–adrenal axis	The consumption of probiotic yogurt or a multispecies probiotic capsule had beneficial effects on mental health parameters in petrochemical workers.	+++
Kato-Kataoka et al., (2016) [102]	Fourth-grade medical students in exam period; *n* = 47; 36.8% ♀	Randomized DBPC/Japan	*Lactobacillus casei Shirota* YIT9029 (LcS) in fermented milk (100 mL); 1 × 10^9^ CFU/mL, 8 w	STAI;HADS;SDS;PSQI	Significant increase of STAI score in both groups before exam compared to baseline but no differences between groups NS differences in the HADS-anxiety, HADS depression, SDS, and PSQI scores either within or between groups NS in cortisol, L-tryptophan, and L-kynurenine levels between groups Logarithmic level of fecal serotonin was significantly higher in the LcS group than in the placebo group at two weeks after the examination	Daily administration of LcS for eight weeks did not affect subjective anxiety	++++
Allen et al., (2016) [104]	Healthy male volunteers under psychological and physiological stressor (SECPT); *n* = 22	Placebo-controlled, repeated-measures, design/Ireland	*Bifidobacterium longum* 1714; 1 × 10^9^ CFU/d; 1 m	PSS-10/EEG; cognitive tasks	Significant increase of anxiety score (STAI) post-stress with placebo. No significant increase under probiotic treatment No differences in cortisol levels between treatments during SECPT Significant enhance of frontal midline mobility (Fz) and reduction in theta power (Cz) post-psychobiotic compared with post-placebo	Consumption of B. *longum* 1714 is associated with reduced stress and improved memory	+++
Messaoudi et al., (2011) [107]	Healthy Caucasian people; *n* = 55; 74.5% ♀	Randomized DBPC/France	*L. helveticus* R0052 and *B. longum* R0175; 9 × 10^9^ CFU/d; 1 m	HSCL-90; HADS, PSS, CCL	HSCL-90: PG significantly changed the global severity index due to improvement in somatization, depression, and anger–hostility HADS: The percentage changes in HADS and HADS-A scores were higher in the PG (*p* < 0.05). No differences in HADS-D between groups PSS: NS differences between groups CCL: NS changes between groups. Significant improve of Problem solving score in PG compared to baseline	*L. helveticus* R0052 and *B. longum* R0175 taken in combination display beneficial psychological effects in healthy human volunteers.	*+++*

Abbreviations. ACQ-R: Anxiety control questionnaire-revised; BAI: Beck anxiety inventory; BDI: Beck depression inventory; CANTAB^30^: Cambridge Neuropsychological Test Automated Battery; CCL: coping checklist; CFQ: Chalder Fatigue Survey; CG: control group; DASS-42: Depression, Anxiety and Stress Scale; DBPC: Randomized Double-Blind, Placebo-Controlled; EEG: electroencephalography; EPDS: Edinburgh Postnatal Depression Scale; GHQ-28: General Health Questionnaire; HADS: Hospital Anxiety and Depression Scale; HAM-A: Hamilton anxiety rating scale; HSCL-90: Hopkins Symptom Checklist; IFN-γ: interferon-gamma; NMR: Negative mood regulation; OSA: Oguri–Shirakawa–Azumi sleep inventory; PANAS: positive and negative affect schedule; PG: probiotic/postbiotic group; POS: psychobiotics oral suspension; PSQI: Pittsburgh Sleep Quality Index; PSS-10: Cohen′s Perceived Stress Scale; PSWQ: Penn state worry questionnaire; RCT: randomized control trial; SDS: Zung Self-Rating Depression Scale; SCFA: short chain fatty acids; SECPT: socially evaluated cold pressor test; STAI: Spielberger State–Trait Anxiety Inventory; STAI6: State Trait Anxiety Inventory 6 item version; TNF-α: tumor necrosis factor alpha; d: days; w:weeks; m: months. NS: non-significant; * *p* < 0.05; ** *p* < 0.01. Quality of evidence grades: high (++++), moderate (+++), low (++). Simbols: ↓: decrease; ↑: increase; ^#^: *S. thermophilus* CNCM I-1630*; L. bulgaricus* CNCM I-1632 *and I-1519; L. lactis subsp. lactis* CNCM I-1631*; L. acidophilus; S. thermophilus; L. plantarum; B. lactis* CNCM I-2494*; L. reuteri* DSM 17938 (1.5 × 10^10^ each); ^§^: *L. acidophilus* LA5 and *B. lactis* BB12 (1 × 107 CFU); ^°^ *L. casei* (3 × 10^3^), *L. acidophilus* (3 × 10^7^), *L. rhamnosus* (7 × 10^9^), *L. bulgaricus* (5 × 10^8^), *B. breve* (2 × 10^10^), *B. longum* (1 × 10^9^), *S. thermophilus* (3 × 10^8^ CFU/g), and 100 mg fructo-oligosaccharide.

**Table 4:** Summary of GRADE evidence profile.

Quality Assessment						No. of Patients	Effect		Quality		Importance
No. of Studies	Design	Risk of Bias	Inconsistency	Indirectness	Imprecision		Ctrl	Relative (95%CI)	Absolute		
Leyrolle (2020) [82]: Composition of the obese BED patients fecal microbiome (no follow-up)											
1	Cross-sectional study	No serious risk of bias	No serious inconsistency	No serious indirectness	No serious imprecision	101 obese subjects	-	OR 0.79–4.4	1.96 ± 4.44 non binge-eaters 0.51 ± 1.03 BED patients 0.04 ± 0.09 non binge-eaters 0.01 ± 0.02 BED patients	Low ++/++++	Important
Breton (2016) [86]: ClpB presence in human plasma (no follow-up)											
1	Case control study	Serious	No serious inconsistency	No serious indirectness	No serious imprecision	66 cases	29	-	1.7–162.7 and 1.4–333 pM	Low ++/++++	Important
Raevuori (2016) [89]: Use of antimicrobials as an indicator for infection prior to the onset of the eating disorder (Five years follow-up)											
1	Case control study	No serious risk of bias	No serious inconsistency	No serious indirectness	No serious imprecision	1592cases	6368	OR 0.9 AN-OR 2.6 BED	71.2% AN (74.3% controls) 89.6% BED (77.6% controls)	Moderate +++/++++	Important
Morita 2015 [62]: Gut dybiosis in patients with AN (no follow-up)											
1	Cross-sectional study	No serious risk of bias	No serious inconsistency	No serious indirectness	No serious imprecision	25AN 14 ANR 11 ANBP	21	AN: the counts of bacteria, *Clostridium coccoides*, *Clostridium leptum*, *Bacteroides fragilis*, *Streptococcus* and *Lactobacillus* were significantly lower *p*< 0.00084	-	Low ++/++++	Important
Borgo (2017) [63]: Microbiota in AN: the triangle between bacterial species, metabolites and psychological test (no follow-up)											
1	Case control study	No serious risk of bias	No serious inconsistency	No serious indirectness	No serious imprecision	15 AN	15	AN: increase of Gram-negative bacteria (*p* = 0.03) AN: affected the composition of microbiota at every taxonomic level, (per-MANOVA; *p* < 0.05)	-	Low ++/++++	Important
Mack (2016) [66]: weitgh gain in AN (no follow-up)											
1	Case control study	No serious risk of bias	No serious inconsistency	No serious indirectness	No serious imprecision	55 AN before WG 44 AN after WG	55 NW	% of relative abundance AN 0.10 (0.05–014) NW 0.01 (0.04–04) Proportions of BCFA: ANT1 11.9 (8.1–17.1) ANT2 13.6 (9.7–18.3) NW 21.1 (15.3–26.7)	-	Low ++/++++	Important
Morkl (2017) [69]											
1	Cross Sectional study	No serious risk of bias	No serious inconsistency	No serious indirectness	No serious imprecision	18 AN 20 athletes (AT), 22 overweight (OW), and 20 obese (OB) women	26	-AT displayed higher level of species richness compared to AN patients (*p* =0.038) and OB participants (*p* = 0.012). OB participants had a significantly lower diversity than OW participants (*p* = 0.047) -Comparative analysis revealed *Coriobacteriaceae* as the only enriched phylotype in AN compared to other entities (LDA score >3.5)	-	Low ++/++++	Important
Armougom (2009) [64]: (no follow-up)											
1	Case control study	No serious risk of bias	No serious inconsistency	No serious indirectness	No serious imprecision	9 AN20 obese	20	-Highly sensitive of *Bacteroides* system (89.89%) and *Firmicutes* (88.89%) and *M. Smithii* (88.89%) -Reduction in the *Bacteroidetes* community in obese patients (*p* < 0.01). Higher *Lactobacillus* species concentration in obese (*p* = 0.0197) or AN (*p* = 0.0332). Higher *M. smithii* in AN (*p* = 0.0171)		Low ++/++++	Important
Hanachi (2018) [67]: (no follow-up)											
1	Case control study	No serious risk of bias	No serious inconsistency	No serious indirectness	No serious imprecision	33 AN	22	-AN: lower alpha-diversity (*p* = 0.03) for Chao1 indexes but not (*p* = 0.203) for Shannon indexes. -Severity of FIDs was strongly correlated with several microbial genera (r = −0.581 belonging to *Peptostreptococcaceae* family; r = 0.392 for *Dialister*, r = 0.444 for *Robinsoniella* and r = 0.488 for *Enterococcus*)		Low ++/++++	Important
Kleiman (2017) [74], Pfleider (2013) [58], Gouba (2014) [59], Prochazkoya (2019) [75], De Clercq (2019) [76], (no follow-up): clinical cases											
5	Clinical case	No serious risk of bias	No serious inconsistency	No serious indirectness	No serious imprecision	Intestinal microbiota in single patients with AN	-	-	-	Very Low +/++++	Less Important
Tran et al., (2019) [100]: effect of probiotics intervention on mental health like anxiety in students; 28 days of follow-up											
1	RDBPC	Medium	No major inconsistencies	High	Medium	Group A: 11; Group B: 13; Group D: 15; Group E: 15	11		Participants with the probiotics intervention as a whole showed a significant decrease in panic anxiety (Mdiff = 0.42, SD = 1.55), and negative affect (Mdiff = 1.79, SD = 6.49)	Low ++/++++	Moderate
Smith-Ryan et al., (2019) [99]: effects of a multi-strain probiotic plus prebiotic on anxiety in female healthcare workers; 6 weeks of follow-up											
1	RDBPC	No serious risk of bias	No major inconsistencies	Medium	Medium	15	18		No significant difference in changes for HADS-A (*p* = 0.621), HADS-D (*p* = 0.506) or CFQ-11 (*p* = 0.773)	Moderate +++/++++	Low
Nishida et al., (2019) [98]: effect of a heat treated probiotic strain on mental conditions in students; 6 months of follow-up											
1	RDBPC	No serious risk of bias	No major inconsistencies	Low	Low	29	31		Significantly reduction of STAI-trait scores in PG compared to CG (−1.9 vs +1.1)	Moderate +++/++++	Critical
Lew et al., (2019) [108]: effects of one probiotic strain in alleviation of stress in stressed adult; 3 months of follow-up											
1	RDBPC	No serious risk of bias	No major inconsistencies	Low	Low	52	51		Reduced scores of stress (mean difference 2.94; 95% CI 0.08 to 5.73; *p* = 0.048), anxiety (mean difference 2.82; 95% CI 0.35 to 5.30; *p* = 0.031) and total score (mean difference 8.04; 95% CI 0.73 to 15.30; *p* = 0.041)	High ++++/++++	Critical
Chong et al., (2019) [109]: effects of one probiotic strain in alleviation of anxiety in stressed adult; 3 months of follow-up											
1	RDBPC	No serious risk of bias	No major inconsistencies	Low	Low	56	55		Reduced scores for anxiety in all subjects versus placebo (*p* = 0.017)	High ++++/++++	Critical
Takada et al., (2017) [101]; Takada et al. (2016) [103] and Kato-Kataoka et al., (2016) [102]: effects of one strain in alleviation of anxiety in healthy 4th year medical students; from 8 to 11 weeks of follow-up											
3	RDBPC	No serious risk of bias	No major inconsistencies	Low	Low	142	139		No differences in STAI scores versus placebo	High ++++/++++	Moderate
Slykerman et al. (2017) [110]: effects of one probiotic strain on postpartum anxiety; from 35 weeks gestation until 6 months if breastfeeding											
1	RDBPC	Serious	No major inconsistencies	Moderate	Moderate	193	187	OR = 0.44 (0.26, 0.73)		Low ++/++++	Low
Kelly et al., (2016) [111]: effects of one probiotic strain in healthy male subjects under the socially evaluated cold pressor test (SECPT); 1 month of follow-up											
1	RPC, cross-over, repeated measures	No serious risk of bias	No major inconsistencies	Low	Moderate	39			No overall effect of treatment phase on Beck Anxiety Inventory (*p* = 0.95), the State Anxiety Inventory (*p* = 0.09) and the Trait Anxiety Inventory (*p* = 0.72)	Moderate +++/++++	Critical
Colica et al., (2017) [106]: effects of a multi-strain probiotic on anxiety in healthy subjects; 3 weeks of follow-up											
1	RCT	Serious	No major inconsistencies	Moderate	High	Group A: 11; Group C: 11	11		Decrease of HAM-A total score (Δ = −5 points). Significant reduction in the number of anxious subjects in probiotic group (*p* = 0.03; Δ% = −39.3%),	Low ++/++++	Low
Mohammadi et al., (2016) [105]: effects of a multi-strain probiotic on anxiety in petrochemical workers; 6 weeks of follow-up											
1	RDBPC	No serious risk of bias	No major inconsistencies	Low	Low	Group A: 25 Group B: 25	20		Significant decrease in DASS score after probiotic intake (from 18.9 ± 3.2 to 9.4± 4.0; change: −9.5 ± 4.3): *p* = 0.006	Moderate +++/++++	Moderate
Allen et al., (2016) [105]: effects of one probiotic strain in healthy male subjects under the socially evaluated cold pressor test (SECPT); 1 month of follow-up											
1	Repeated measures, placebo-controlled	No serious risk of bias	No major inconsistencies	Low	Low	22			No significant differences of STAI scores pre and post stressor	Moderate +++/++++	Critical
Mesaoudi et al., (2011) [107]: effects of a multi-strain probiotic on anxiety in healthy subjects; 1 month of follow-up											
1	RDBPC	No serious risk of bias	No major inconsistencies	Moderate	Low	26	29		Significant decrease decrease in HADS-A score in probiotic group. Change between baseline and follow-up (%): median: 36.9% (20–50%; IQ-SQ)	Moderate +++/++++	Moderate

Quality of evidence grades: high (++++), moderate (+++), low (++), very low (+).

## Supplementary Materials

The following are available online at https://www.mdpi.com/1422-0067/22/5/2351/s1: Supplemental Material S1: Search Methodology.

## Abbreviations

α-MSH	α-Melanocyte stimulating hormone
AD	Anti-depressant
BBB	Blood-brain barrier
BDNF	Brain-derived neurotrophic factor
BED	Binge eating disorder
BEGIN	Begin Eating Genetics Initiative
BN	Bulimia nervosa
BMI	Body mass index
BN	Bulimia nervosa
BZ	Benzodiazepine
ClpB	Caseinolyitic protease b
CLDN1	Claudin 1
CNS	Central nervous system
DASS-42	Depression Anxiety Stress Scale 42
ED	Eating disorder
EEG	Electroencephalography
EPDS	Edinburgh Postnatal Depression Scale
FMT	Fecal microbiota transplantation
GABA	Gamma aminobutyric acid
GAS	Generalized anxiety disorders
GHQ	General Health Questionnaire
HADS-A	Hospital Anxiety and Depression Scale-anxiety
HAM-A	Hamilton Rating Scale for Anxiety
HPA	Hypothalamic-pituitary-adrenal
IDO	Indoleamine 2,3-dioxygenase
IFN-γ	Interferon-γ
IL	Interleukin
KEGG	Kyoto Encyclopedia of Genes and Genomes annotation
LPS	Lipopolysaccharides
OCD	Obsessive-compulsive disorder
OUT	Operational taxonomic unit
PSQI	Pittsburgh Sleep Quality Index
PSS	Perceived Stress Scale
PVN	Para-ventricular nucleus
RCT	Randomized clinical trial
SCFA	Short-chain fatty acids
SDS	Self-rating Depression Scale
SGBs	Species-level genome bins
STAI	State Trait Anxiety Inventory
TLR	Toll-like receptor
TNF-α	Tumor necrosis factor-α
