# Supplementary material for: Effects of Microbiota Imbalance in Anxiety and Eating Disorders: Probiotics as Novel Therapeutic Approaches
Source: Int J Mol Sci. 2021 Feb 26;22(5):2351. doi: 10.3390/ijms22052351 (PMC7956573; doi:10.3390/ijms22052351)
Supplement: Supplementary file 1 [file ijms-22-02351-s001.pdf]

Supplemental Material S1

Search methodology

Section 2.1. MeSH terms (PubMed) and search: (((“faecally”[All Fields] OR “fecally”[All Fields] OR “fecals”[All Fields] OR “feces”[MeSH Terms] OR “feces”[All Fields] OR “faecal”[All Fields] OR “fecal”[All Fields]) AND (“microbiota”[MeSH Terms] OR “microbiota”[All Fields] OR “microbiotas”[All Fields] OR “microbiota s”[All Fields] OR “microbiotae”[All Fields])) OR (“gastrointestinal microbiome”[MeSH Terms] OR (“gastrointestinal”[All Fields] AND “microbiome”[All Fields]) OR “gastrointestinal microbiome”[All Fields] OR (“gut”[All Fields] AND “microbiota”[All Fields]) OR “gut microbiota”[All Fields])) AND ((“drugs, generic”[MeSH Terms] OR (“drugs”[All Fields] AND “generic”[All Fields]) OR “generic drugs”[All Fields] OR “generic”[All Fields] OR “family characteristics”[MeSH Terms] OR (“family”[All Fields] AND “characteristics”[All Fields]) OR “family characteristics”[All Fields] OR “generation”[All Fields] OR “generations”[All Fields] OR “general”[All Fields] OR “general s”[All Fields] OR “generalisability”[All Fields] OR “generalisable”[All Fields] OR “generalisation”[All Fields] OR “generalization, psychological”[MeSH Terms] OR (“generalization”[All Fields] AND “psychological”[All Fields]) OR “psychological generalization”[All Fields] OR “generalization”[All Fields] OR “generalisations”[All Fields] OR “generalise”[All Fields] OR “generalised”[All Fields] OR “generalises”[All Fields] OR “generalisibility”[All Fields] OR “generalising”[All Fields] OR “generalities”[All Fields] OR “generality”[All Fields] OR “generalizability”[All Fields] OR “generalizable”[All Fields] OR “generalizations”[All Fields] OR “generalize”[All Fields] OR “generalized”[All Fields] OR “generalizes”[All Fields] OR “generalizing”[All Fields] OR “generally”[All Fields] OR “generals”[All Fields] OR “generate”[All Fields] OR “generated”[All Fields] OR “generates”[All Fields] OR “generating”[All Fields] OR “generation s”[All Fields] OR “generational”[All Fields] OR “generative”[All Fields] OR “generatively”[All Fields] OR “generativity”[All Fields] OR “generator”[All Fields] OR “generator s”[All Fields] OR “generators”[All Fields] OR “generically”[All Fields] OR “genericity”[All Fields] OR “generics”[All Fields]) AND (“anxiety disorders”[MeSH Terms] OR (“anxiety”[All Fields] AND “disorders”[All Fields]) OR “anxiety disorders”[All Fields] OR (“anxiety”[All Fields] AND “disorder”[All Fields]) OR “anxiety disorder”[All Fields])). Filters: Article types: case reports, classical article, clinical study, clinical trial, comparative study, controlled clinical trial, multicenter study, observational study, randomized controlled trial; Publication dates: 2004-2019(included); Species: humans; Languages: English;

Section 2.2. MeSH terms (PubMed) and search: “(“gastrointestinal microbiome”[MeSH Terms] OR (“gastrointestinal”[All Fields] AND “microbiome”[All Fields]) OR “gastrointestinal microbiome”[All Fields] OR (“gut”[All Fields] AND “microbiota”[All Fields]) OR “gut microbiota”[All Fields] OR (“dysbiosis”[MeSH Terms] OR “dysbiosis”[All Fields] OR “dysbioses”[All Fields]) OR (“microbiome s”[All Fields] OR “microbiomic”[All Fields] OR “microbiomics”[All Fields] OR “microbiota”[MeSH Terms] OR “microbiota”[All Fields] OR “microbiome”[All Fields] OR “microbiomes”[All Fields])) AND (“anorexia nervosa”[MeSH Terms] OR (“anorexia”[All Fields] AND “nervosa”[All Fields]) OR “anorexia nervosa”[All Fields])” Filters: Article types: classical article, clinical study, clinical trial, comparative study, controlled clinical trial, journal article, multicenter study, observational study, randomized controlled trial; Publication dates: 2008-2020(included); Species: humans; Languages: English.

Section 2.3. MeSH terms (PubMed) and search for Bulimia Nervosa “ (((“Microbiota”[Mesh]) OR (“Gastrointestinal Microbiome”[Mesh])) AND ((“Bulimia”[Mesh]) OR (“Bulimia Nervosa”[Mesh]))) “; for Binge-Eating Disorder “ (((“Microbiota”[Mesh]) OR (“Gastrointestinal Microbiome”[Mesh])) AND “Binge-Eating Disorder”[Mesh]))”. Filters: Article types: classical article, clinical study, clinical trial, comparative study, controlled clinical trial, journal article, multicenter study, observational study, randomized controlled trial; Publication dates: 2008-2020 (included); Languages: English.

Section 2.4. MeSH terms (PubMed) and search “ ((“fecal microbiota transplantation”[Mesh]) OR (“probiotics”[Mesh]) OR (“prebiotics”[Mesh]) OR (“anti-bacterial agents”[Mesh])) AND “feeding and Eating Disorders”[Mesh] “. (“psychobiotic”[All Fields] OR “psychobiotics”[All Fields] OR (“probiotic s”[All Fields] OR “probiotical”[All Fields] OR “probiotics”[MeSH Terms] OR “probiotics”[All Fields] OR “probiotic”[All Fields]) OR (“prebiotically”[All Fields] OR “prebiotics”[MeSH Terms] OR “prebiotics”[All Fields] OR “prebiotic”[All Fields]) OR (“symbiote”[All Fields] OR “symbiotes”[All Fields] OR “symbiotic”[All Fields] OR “symbiotically”[All Fields] OR “symbiotics”[All Fields]) OR (“fecal microbiota transplantation”[MeSH Terms] OR (“fecal”[All Fields] AND “microbiota”[All Fields] AND “transplantation”[All Fields]) OR “fecal microbiota transplantation”[All Fields] OR (“fecal”[All Fields] AND “transplantation”[All Fields]) OR “fecal transplantation”[All Fields]) OR (“postbiotic”[All

Fields] OR “postbiotics”[All Fields])) AND (“GAD”[All Fields] OR (“anxiety”[MeSH Terms] OR “anxiety”[All Fields] OR “anxieties”[All Fields] OR “anxiety s”[All Fields])). Filters: classical article, clinical study, clinical trial, comparative study, controlled clinical trial, journal article, multicenter study, observational study, randomized controlled trial; Publication dates: 2000-2020 (included); Languages: English.
